# Heterologous versus homologous boosting elicits qualitatively distinct, BA.5–cross-reactive T cells in transplant recipients

**DOI:** 10.1172/jci.insight.168470

**Published:** 2023-05-22

**Authors:** Elizabeth A. Thompson, Wabathi Ngecu, Laila Stoddart, Trevor S. Johnston, Amy Chang, Katherine Cascino, Jennifer L. Alejo, Aura T. Abedon, Hady Samaha, Nadine Rouphael, Aaron A.R. Tobian, Dorry L. Segev, William A. Werbel, Andrew H. Karaba, Joel N. Blankson, Andrea L. Cox

**Affiliations:** 1 Department of Medicine,; 2 Bloomberg~Kimmel Institute for Cancer Immunotherapy, and; 3 Department of Surgery, Johns Hopkins University School of Medicine, Baltimore, Maryland, USA.; 4 Hope Clinic, Infectious Diseases Division, Emory University, Decatur, Georgia, USA.; 5 Department of Oncology and; 6 Department of Pathology, Johns Hopkins University School of Medicine, Baltimore, Maryland, USA.; 7 Department of Surgery, New York University Grossman School of Medicine, New York, New York, USA.; 8 Department of Molecular Microbiology and Immunology, Johns Hopkins University Bloomberg School of Public Health, Baltimore, Maryland, USA.

**Keywords:** COVID-19, Vaccines, Adaptive immunity, Organ transplantation, T cells

## Abstract

**Background:**

The SARS-CoV-2 Omicron BA.5 subvariant escapes vaccination-induced neutralizing antibodies because of mutations in the spike (S) protein. Solid organ transplant recipients (SOTRs) develop high COVID-19 morbidity and poor Omicron variant recognition after COVID-19 vaccination. T cell responses may provide a second line of defense. Therefore, understanding which vaccine regimens induce robust, conserved T cell responses is critical.

**Methods:**

We evaluated anti-S IgG titers, subvariant pseudo-neutralization, and S-specific CD4^+^ and CD8^+^ T cell responses from SOTRs in a national, prospective, observational trial (*n* = 75). Participants were selected if they received 3 doses of mRNA (homologous boosting) or 2 doses of mRNA followed by Ad26.COV2.S (heterologous boosting).

**Results:**

Homologous boosting with 3 mRNA doses induced the highest anti-S IgG titers. However, antibodies induced by both vaccine regimens demonstrated lower pseudo-neutralization against BA.5 compared with the ancestral strain. In contrast, vaccine-induced S-specific T cells maintained cross-reactivity against BA.5 compared with ancestral recognition. Homologous boosting induced higher frequencies of activated polyfunctional CD4^+^ T cell responses, with polyfunctional IL-21^+^ peripheral T follicular helper cells increased in mRNA-1273 compared with BNT162b2. IL-21^+^ cells correlated with antibody titers. Heterologous boosting with Ad26.COV2.S did not increase CD8^+^ responses compared to homologous boosting.

**Conclusion:**

Boosting with the ancestral strain can induce cross-reactive T cell responses against emerging variants in SOTRs, but alternative vaccine strategies are required to induce robust CD8^+^ T cell responses.

**Funding:**

Ben-Dov Family; NIH National Institute of Allergy and Infectious Diseases (NIAID) K24AI144954, NIAID K08AI156021, NIAID K23AI157893, NIAID U01AI138897, National Institute of Diabetes and Digestive and Kidney Diseases T32DK007713, and National Cancer Institute 1U54CA260492; Johns Hopkins Vice Dean of Research Support for COVID-19 Research in Immunopathogenesis; and Emory COVID-19 research repository.

## Graphical abstract

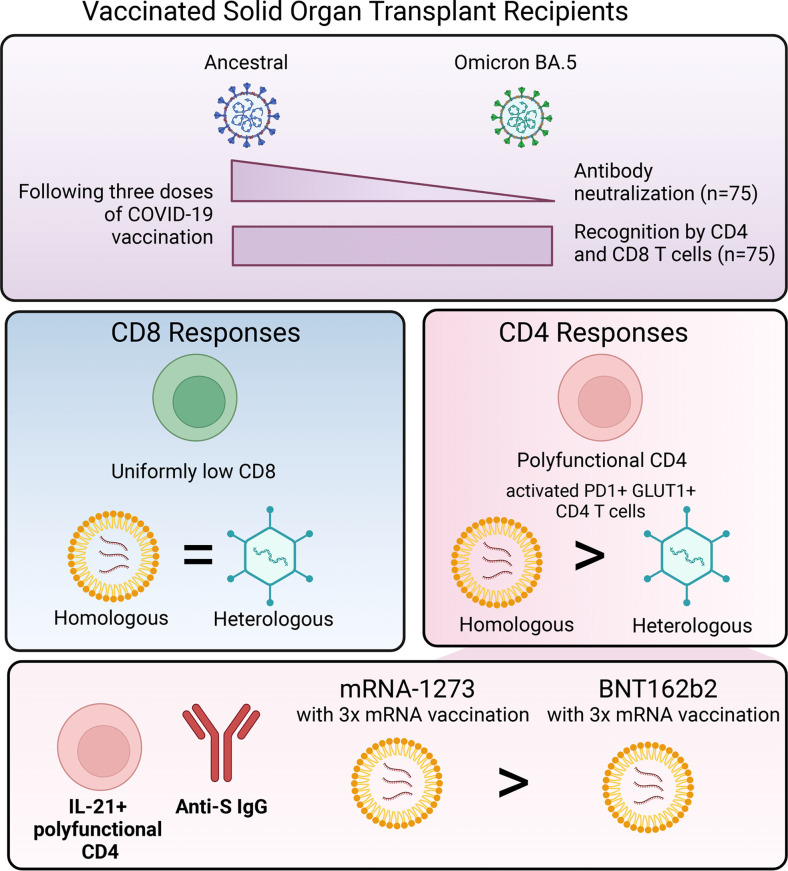

## Introduction

Globally, over 11 billion doses of vaccines against SARS-CoV-2 have been delivered. However, vaccines have not provided immunocompromised populations the same level of protection as healthy individuals (1–4). In particular, solid organ transplant recipients (SOTRs) demonstrate the lowest levels of anti-spike (anti-S) antibody seroconversion following vaccination and develop higher rates of clinically significant breakthrough infection (5–11). As SARS-CoV-2 continues to evolve, vaccine-induced antibodies (Abs) have demonstrated progressively diminished capacity to neutralize more recent variants of concern (VOCs), which may disproportionately affect immunocompromised populations such as SOTRs, even following repeated vaccine dosing (12–15).

In November of 2021, a novel VOC designated Omicron B.1.1.529 (B1) was first identified in South Africa and spread rapidly throughout the world (16). In contrast to earlier VOCs, Omicron demonstrated substantial mutations in the SARS-CoV-2 S protein, resulting in partial or complete viral escape from vaccine-induced Abs (17). Since the emergence of BA.1, several subvariants have arisen, with Omicron BA.4 and BA.5 variants quickly becoming prevalent globally. Several reports have demonstrated reduced Ab neutralization of the BA.4 and BA.5 variants because of the substantial mutations within the S protein (18, 19). However, T cell responses in immunocompetent individuals are more cross-reactive against Omicron VOC than Abs are, and T cells continue to recognize early Omicron subvariants. Preclinical nonhuman primate models have demonstrated a protective effect of CD8^+^ T cells against SARS-CoV-2 infection following vaccination (23). Therefore, conserved T cell responses, particularly CD8^+^ responses, may provide an important second line of protection against current and emerging VOCs in both healthy and immunosuppressed individuals (24).

mRNA vaccines have been the most widely utilized platform for COVID-19 vaccination in the United States. While these platforms generate robust Ab titers and CD4^+^ T cell responses, they induce less robust CD8^+^ responses (25). In contrast, adenoviral (Ad) vectors have long been known to induce CD8^+^ T cell responses following vaccination against other pathogens (26, 27). Efforts to improve immunogenicity have further demonstrated that heterologous boosting strategies utilizing Ad vectors can further enhance the development of CD8^+^ T cell responses against other pathogens (28–30). However, there has been limited comprehensive investigation of heterologous boosting strategies in healthy or immunocompromised individuals, including SOTRs, who are on maintenance immunosuppression to prevent organ rejection. Limited data in healthy individuals have shown varying influence on T cell responses following heterologous boosting depending on the specific Ad vector used, timing/order of vaccinations, and number of Ad vector doses given (31–33). Therefore, we sought to investigate BA.5-reactive T cell responses in SOTRs and the impact of boosting with Ad vectored vaccines compared with mRNA. Given the prevalence of mRNA vaccination in the United States, we focused specifically on delivering a third dose of mRNA or Ad vector following 2 primary doses of mRNA vaccination as a strategy to enhance T cell and Ab responses.

## Results

### Cohort description.

Plasma and peripheral blood mononuclear cells (PBMCs) from a national, prospective, observational cohort evaluating SARS-CoV-2 vaccination in SOTRs were collected to assess humoral and cellular responses before and after a third dose of SARS-CoV-2 vaccination (*n* = 75, Table 1). Participants from the observational cohort were selected for this study based on those who received 2 prior doses of mRNA vaccine containing the ancestral spike sequence (Moderna mRNA-1273 or Pfizer/BioNTech BNT162b2) and subsequently received either a homologous third dose of mRNA vaccine or heterologous boost with the Ad26.COV2.S (Ad26) viral vector–based vaccine (Johnson and Johnson/Janssen JNJ-78436735). Choice of vaccine was at the discretion of individual participants and their transplant providers. Anti-S IgG titers and T cell responses were measured approximately 2 weeks after a third dose. Analysis was also performed on 4 subgroups depending on mRNA formulation (mRNA-1273 vs. BNT162b2) of the first 2 doses and whether the third dose was mRNA or Ad26 based (Supplemental Table 1; supplemental material available online with this article; https://doi.org/10.1172/jci.insight.168470DS1). Participants with self-reported previous SARS-CoV-2 infection or detection of anti-nucleocapsid Abs above the positive threshold were excluded from analysis. Given recent data demonstrating blunting of anti-nucleocapsid Ab induction from breakthrough infection following vaccination, we may not have excluded all participants with asymptomatic infection using anti-nucleocapsid screening (34). There were no statistically significant differences between the groups in terms of transplanted organ or immunosuppressive regimen between the 2 arms; however, age and time since transplant were overall lower in the mRNA group (Table 1). We therefore performed subsequent analysis adjusting for key demographic and transplant factors known be associated with vaccine response among SOTRs, as described below (35–38).

### Vaccine-induced Abs demonstrate reduced pseudo-neutralization of the Omicron BA.5 VOC.

**Figure 1:**
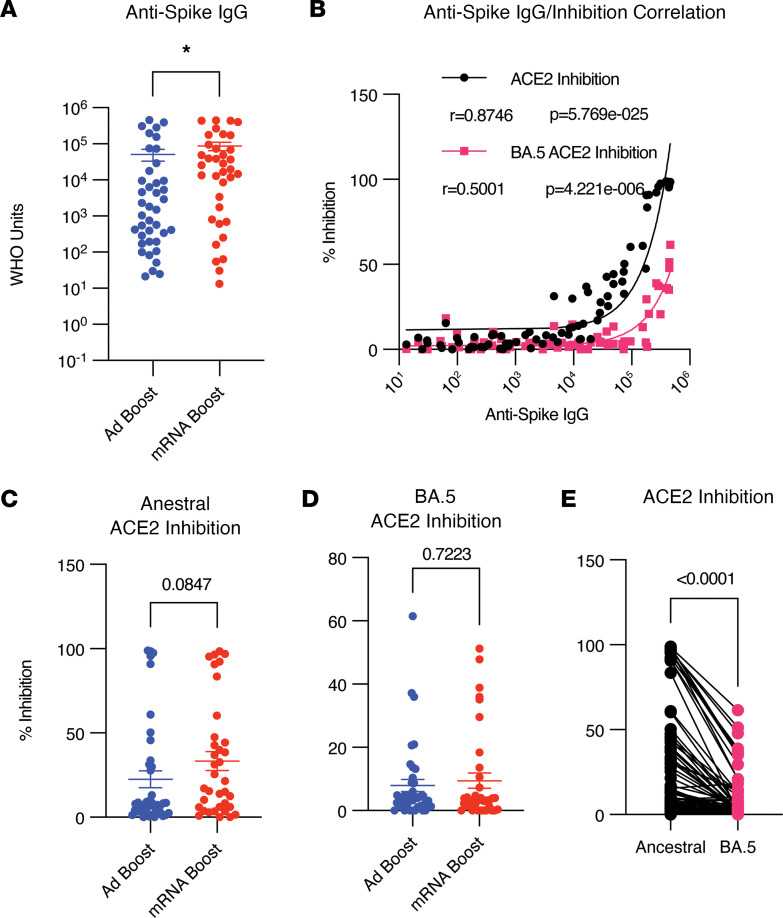
Increased Ab titers following mRNA boost with loss of BA.5 recognition regardless of regimen. Plasma Ab responses were evaluated in participants who received 2 doses of an mRNA COVID-19 vaccine followed by adenoviral vector boost (Ad boost, blue, *n* = 40) or a third mRNA dose (mRNA boost, red, *n* = 35). Samples were collected approximately 2 weeks following the third dose. (**A**) Anti-spike (Anti-S) IgG titers as determined by Meso Scale Diagnostics (MSD) research assay. Significance tested using the Mann-Whitney *U* test. (**B**) Correlation between anti-S IgG and pseudo-neutralization MSD ACE2 binding inhibition assay against the ancestral WA-1 S protein or the BA.5 S protein. Significance tested using nonparametric Spearman correlation. (**C** and **D**) Comparison of ancestral ACE2 inhibition (**C**) or BA.5 ACE2 inhibition (**D**). Significance tested using the Mann-Whitney *U* test. (**E**) Sample matched comparison of ACE2 inhibition using ancestral or BA.5 S protein. Significance tested using Wilcoxon matched pairs signed-rank test. *P < 0.05. All data shown as mean ± SEM with each dot representing 1 individual.

In studies of the general population, COVID-19 vaccination strategies using only homologous mRNA formulations have demonstrated increased Ab titers compared with either homologous Ad vectors or heterologous strategies containing Ad vectors (32, 39–41). Within this cohort of SOTRs, we confirmed that individuals who received an mRNA boost compared with an Ad boost demonstrated significantly higher anti-S IgG binding titers (Figure 1A). Comparing the 2 mRNA vaccines, individuals receiving 3 doses of mRNA-1273 had the highest titers within this cohort (Supplemental Figure 1). To assess Ab functionality, we used an angiotensin-converting enzyme 2 (ACE2) inhibition assay as a surrogate of viral neutralizing capacity. This assay, when utilized in vaccinated SOTRs, shows strong correlation with live virus-neutralizing Abs, particularly above 20% ACE2 inhibition (5, 42). Although anti-S IgG titers positively correlated with ACE2 inhibition of both the ancestral strain and the BA.5 VOC (Figure 1B), there was a significant decrease in BA.5 pseudo-neutralization versus ancestral strain (Figure 1, C–E). Despite higher titers induced by homologous boosting, most vaccine recipients across all groups failed to mount BA.5-neutralizing activity (Figure 1, C–E). Together, these data demonstrate that 3 homologous mRNA vaccine doses induced higher ancestral S-binding Ab titers than following heterologous Ad boosting in immunosuppressed SOTRs, but the majority of participants did not demonstrate neutralizing capacity against Omicron BA.5, likely due to viral evolution and immune escape.

### CD4^+^ T cells maintain cross-reactivity against BA.5, with higher responses following mRNA boost.

**Figure 2:**
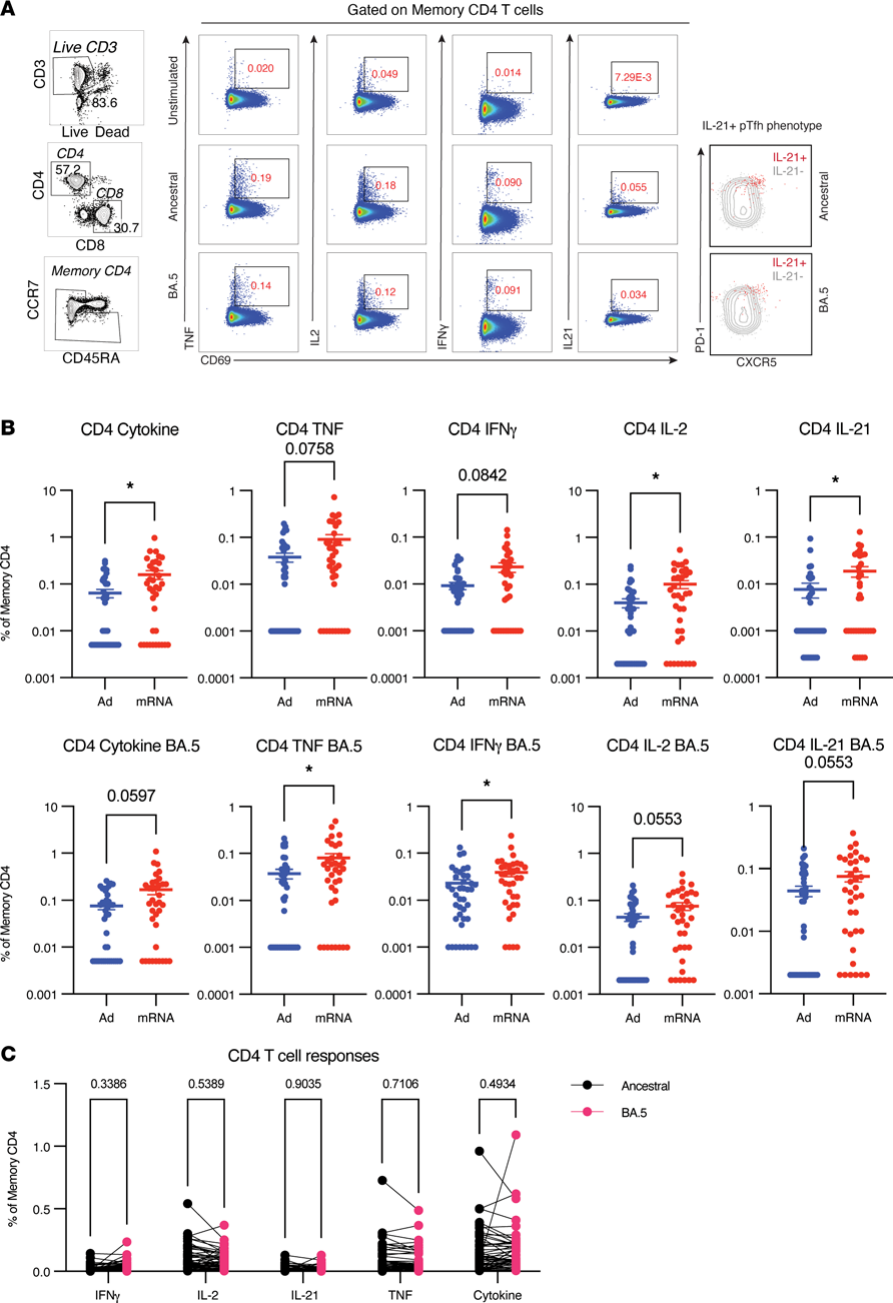
CD4^+^ T cells maintain cross-reactivity against BA.5, with higher responses following mRNA boost. Peripheral blood mononuclear cells (PBMCs) were stimulated overnight with overlapping peptides (15-mers overlapping by 11) against ancestral or BA.5 spike (S) protein. S protein–specific CD4^+^ T cell responses were evaluated in participants who received 2 doses of an mRNA COVID-19 vaccine followed by adenoviral vector boost (Ad boost, blue, *n* = 40) or a third mRNA dose (mRNA boost, red, *n* = 35). Samples were collected approximately 2 weeks following the third dose and run in 2 batches with participants evenly distributed between both batches. (**A**) Representative gating for CD4^+^ T cell responses, including phenotype of IL-21^+^ cells and relation to PD-1^+^CXCR5^+^ expression that defines peripheral T follicular helper (pTfh) cells. PD-1, programmed death 1. (**B**) Frequency of memory CD4^+^ T cells producing any cytokine (TNF, IFN-γ, IL-2, or IL-21) or each individual cytokine. All values are with unstimulated DMSO-only control levels subtracted. Samples with negative or 0 values were converted to the lowest detected value for visualization purposes. Significance tested using Mann-Whitney *U* test. (**C**) Sample paired comparison of CD4^+^ responses recalled by ancestral or BA.5 S peptides. Significance tested using Wilcoxon matched pairs signed-rank test. *P < 0.05. All data shown as mean ± SEM with each dot representing 1 individual.

To investigate vaccine-induced S-specific T cells, PBMCs were stimulated overnight with overlapping S peptides from the ancestral (WA-1/vaccine strain) or BA.5 strains and evaluated for the production of interferon-γ (IFN-γ), interleukin-2 (IL-2), tumor necrosis factor (TNF), or IL-21, or the ability to generate any one of these cytokines (cytokine) (Figure 2A). CD4^+^ T cell responses showed trending or significantly higher production of all cytokines following mRNA boosting as compared with Ad vector boosting (Figure 2B). Total S-specific CD4^+^ T cells (cytokine^+^) were still significantly higher in the homologous mRNA boosting group compared with heterologous boosting, even after adjusting for multiple factors associated with vaccine response in SOTRs, including age ≥ 65 years old, ≤5 years posttransplant, liver-only transplant recipient, anti-metabolite therapy, and belatacept use (Supplemental Table 2). Interestingly, following 3 doses of mRNA vaccines, SOTRs demonstrated comparable CD4^+^ T cell responses to healthy controls receiving 2 doses of BNT162b2 (Supplemental Figure 2). Compared with healthy controls following either 2 or 3 doses of mRNA vaccination, SOTRs receiving 3 doses of mRNA vaccines demonstrated a higher IL-2^+^CD4^+^ T cell response and generated lower levels of IFN-γ. Although healthy individuals saw a further boost in CD4^+^ T cell responses following a third dose, these data support the notion that SOTRs can mount responses similar to those of healthy individuals when they are provided additional doses.

Of particular interest was the increase in IL-21^+^CD4^+^ T cell responses following homologous mRNA boosting. Peripheral T follicular helper (pTfh) cells are a subset of CD4^+^ T cells that are transiently found in the periphery following vaccination or infection and robustly correlate with induction of Ab responses (43–45). pTfh cells are often enumerated using a combination of surface markers directly ex vivo; however, they can also be identified as IL-21–producing CD4^+^ T cells following an antigen recall assay (46). It has been shown that antigen-specific IL-21–producing CD4^+^ T cells found in the periphery are transcriptionally similar to Tfh cells found in the lymph node, which are known to be critical drivers of the germinal center reaction and provide support to B cells (46). In SOTRs, CD4^+^ T cells producing IL-21 following stimulation expressed high levels of PD-1 and CXCR5, consistent with a pTfh phenotype (Figure 2A). However, given that SOTRs’ immunosuppressive regimens are primarily designed to inhibit T cell–mediated organ rejection, we thought it critical to assess pTfh cell functionality via the ability of T cells to secrete cytokines in response to S peptides, instead of exclusively evaluating surface expression markers. Homologous mRNA boosting induced significantly higher IL-21^+^ pTfh cells (Figure 2B), consistent with a role supporting B cell development and the higher anti-S IgG levels observed in this immunization group. Again, IL-21^+^ pTfh cells were increased following homologous mRNA boosting, even after adjusting for multiple variables (Supplemental Table 2). Consistent with 3 doses of mRNA-1273 inducing the highest Ab titers, this vaccine regimen also induced higher levels of S-specific CD4^+^ T cells (Supplemental Figure 3A). In stark contrast to Ab responses, there was no significant difference in the frequency of CD4^+^ T cell responses recognizing BA.5 epitopes compared to the ancestral strain (Figure 2C). Therefore, homologous mRNA boosting led to an enhanced CD4^+^ T cell response with conserved recognition of ancestral and BA.5 viral peptides, and mRNA-1273 induced increased CD4^+^ T cell responses versus BNT162b2 that corresponded with higher Ab titers.

### CD8^+^ T cell responses are low following vaccination but maintain cross-reactivity against BA.5.

**Figure 3:**
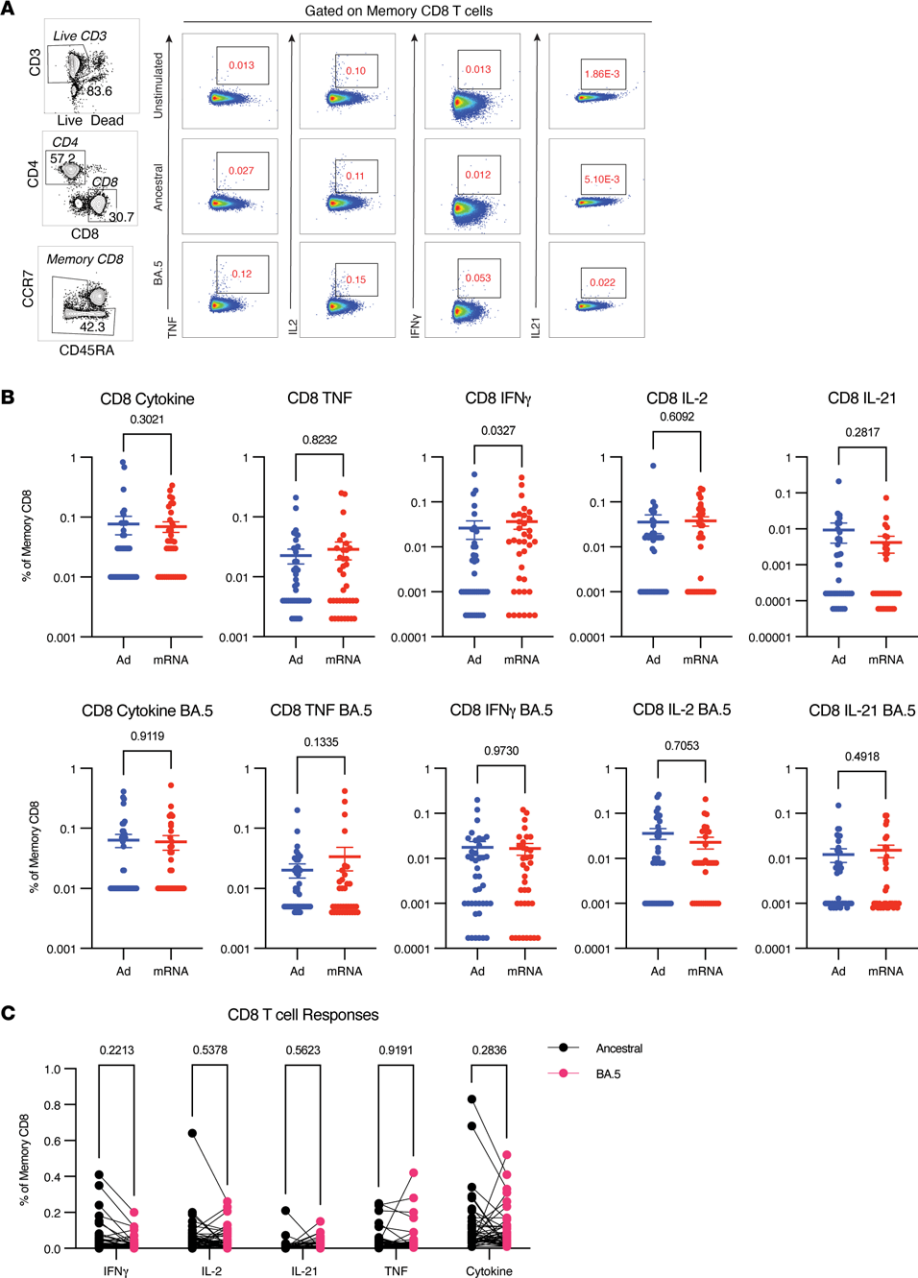
CD8^+^ T cell responses are low following vaccination but maintain cross-reactivity against BA.5. Peripheral blood mononuclear cells (PBMCs) were stimulated overnight with overlapping peptides (15-mers overlapping by 11) against ancestral or BA.5 spike (S) protein. S protein–specific CD8^+^ T cell responses were evaluated in participants who received 2 doses of an mRNA COVID-19 vaccine followed by adenoviral vector boost (Ad boost, blue, *n* = 40) or a third mRNA dose (mRNA boost, red, *n* = 35). Samples were collected approximately 2 weeks following the third dose and run in 2 batches with participants evenly distributed between both batches. (**A**) Representative gating for CD8^+^ T cell responses. (**B**) Frequency of memory CD8^+^ T cells producing any cytokine (TNF, IFN-γ, IL-2, or IL-21) or each individual cytokine. All values are with unstimulated DMSO-only control levels subtracted. Samples with negative or 0 values were converted to the lowest detected value for visualization purposes. Significance tested using Mann-Whitney *U* test. (**C**) Sample paired comparison of CD8 responses recalled by ancestral or BA.5 S peptides. Significance tested using Wilcoxon matched pairs signed-rank test. Significance tested using Wilcoxon matched pairs signed-rank test. All data shown as mean ± SEM with each dot representing 1 individual.

In the setting of reduced Ab neutralization of new VOCs, cross-reactive CD8^+^ T cell responses may provide a second line of defense as the virus continues to accumulate mutations in the S protein. Previous vaccine candidates have demonstrated that Ad-based vectors induce robust CD8^+^ responses and therefore may provide additional Ab-independent protection (26, 27). However, CD8^+^ responses have not been well characterized in SOTRs following different vaccination strategies. Within the SOTR cohort, the overall frequency of cytokine-producing CD8^+^ T cells upon ancestral or BA.5 stimulation was low and often failed to rise above background levels, regardless of vaccine regimen (Figure 3, A and B). In general, there were no significant differences between individuals receiving homologous mRNA boosting or heterologous Ad boosting, despite preclinical data predicting otherwise. Adjusting for multiple clinical and transplant variables did not reveal a significant association between vaccine sequence and CD8^+^ T cell response (Supplemental Table 2). Interestingly, cytokine production from CD8^+^ T cells was also low in healthy controls following 2 or 3 doses of BNT162b2, indicating mRNA-based vaccines induce better CD4^+^ than CD8^+^ T cell responses in both SOTRs and healthy controls (Supplemental Figure 2). However, in contrast to Ab neutralizing activity, there was again no significant difference in recognition of BA.5 epitopes compared to those of the ancestral strain (Figure 3C).

### Increased polyfunctional CD4^+^ T cell responses following mRNA boost.

**Figure 4:**
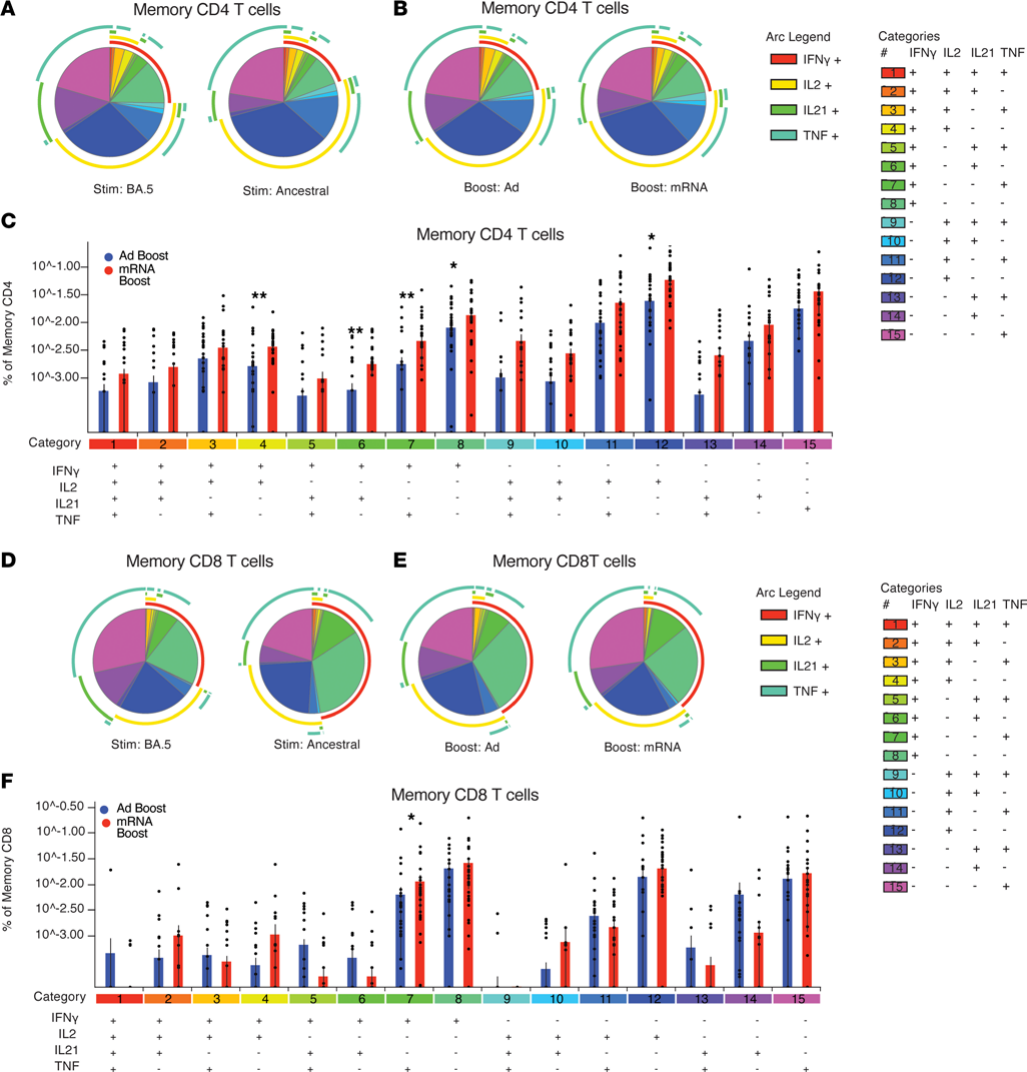
Increased polyfunctional CD4^+^ T cells following mRNA boost. Spike protein–specific T cell responses were evaluated in participants who received 2 doses of an mRNA COVID-19 vaccine followed by adenoviral vector boost (Ad boost, blue, *n* = 40) or a third mRNA dose (mRNA boost, red, *n* = 35). Polyfunctionality of the memory CD4^+^ or CD8 T cell responses. Pie charts show the fraction of total cytokine response comprising any combination of IFN-γ, IL-2, TNF, or IL-21. Pie arcs show the proportion making each cytokine as annotated. (**A**) Comparison of CD4^+^ response to ancestral or BA.5 peptides, regardless of boosting regimen. (**B**) Comparison of CD4^+^ response against BA.5 peptides, stratified by boosting regimen. (**C**) Overview of CD4^+^ response against BA.5 peptides, with percentage of total memory CD4^+^ T cells shown for individual polyfunctional categories. Significance tested using the Wilcoxon ranked test as calculated in SPICE v6. (**D**) Comparison of CD8^+^ response to ancestral or BA.5 peptides, regardless of boosting regimen. (**E**) Comparison of CD8^+^ response against BA.5 peptides, segregated by boosting regimen. (**F**) Overview of CD8^+^ response against BA.5 peptides with percentage of total memory CD8^+^ T cells shown for individual polyfunctional categories. Significance tested using the Wilcoxon ranked test as calculated in SPICE v6.

Polyfunctional T cells, i.e., having the ability to produce more than one cytokine, have been shown to improve protection following vaccination against influenza, cytomegalovirus, and Leishmania infections (47–51). Therefore, we evaluated the polyfunctionality of T cell responses against S peptides following homologous or heterologous boosting strategies. There were no significant differences in polyfunctionality induced by the ancestral or BA.5 peptides, regardless of vaccine regimen (Figure 4, A and D). Therefore, subsequent analysis focused on BA.5-specific responses, as this strain and its sublineages remain in circulation. In response to the BA.5 peptides, homologous boosting induced higher levels of polyfunctional CD4^+^ T cells compared with heterologous boosting (Figure 4, B and C). CD4^+^ T cells producing IFN-γ in combination with any other cytokine (category 4, 6, 7, 8) or IL-2 alone (category 12) were significantly higher in patients receiving an mRNA boost (Figure 4C). There were few differences in polyfunctional CD8^+^ T cells induced following homologous versus heterologous boosting, consistent with limited CD8^+^ T cell responses overall (Figure 4, E and F). However, there were significantly higher IFN-γ^+^TNF^+^CD8^+^ T cells induced by mRNA boosting (Figure 4, E and F, category 7).

**Figure 5:**
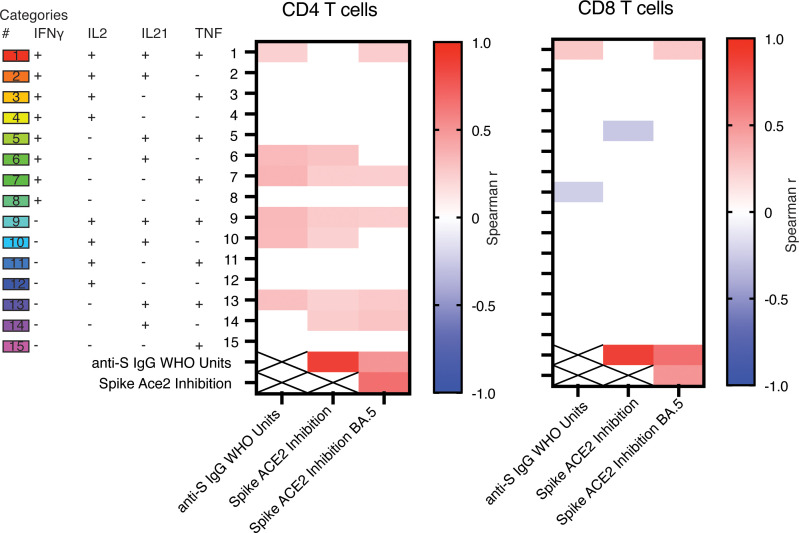
Polyfunctional CD4^+^ T cells correlate with Ab titers. Spike-specific T cell responses were evaluated in participants who received 2 doses of an mRNA COVID-19 vaccine followed by adenoviral vector boost (Ad boost, blue, *n* = 40) or a third mRNA dose (mRNA boost, red, *n* = 35). Polyfunctionality of the memory CD4^+^ or CD8^+^ T cell responses was calculated regardless of boosting regimen. Multivariate correlation of polyfunctional CD4^+^ or CD8^+^ categories with anti-S IgG titers and ACE2 inhibition. Correlation tested using nonparametric Spearman test. Values with nonsignificant correlation (*P* > 0.05) had Spearman coefficient changed to 0.

To understand if polyfunctional T cells correlated with Ab titers and neutralization, we performed multivariate correlative analysis of all CD4^+^ and CD8^+^ T cell cytokine-producing subsets (Figure 5). In general, more CD4^+^ T cell subsets demonstrated a positive correlation with Ab titers and variant pseudo-neutralization, and subsets that contained IL-21^+^CD4^+^ T cells showed the highest positive correlation. Given the increased Ab titers following 3 doses of mRNA-1273 compared with BNT162b2, but limited differences in CD4^+^ T cell responses, we evaluated polyfunctionality induced by these 2 vaccine platforms. mRNA-1273 induced significantly higher levels of polyfunctional T cells compared with BNT162b2, particularly subsets containing IL-21 production (Supplemental Figure 4). Together, these data support that despite immunosuppression, polyfunctional CD4^+^ T cells can be induced by vaccination in some SOTRs. The correlation of polyfunctional pTfh cells with Ab titers also highlights a likely important role of IL-21^+^ pTfh cells in supporting Ab responses in SOTRs.

### Homologous mRNA boost induces qualitatively different CD4^+^ T cells with increased metabolic activity, cytokine production, and memory phenotype.

**Figure 6:**
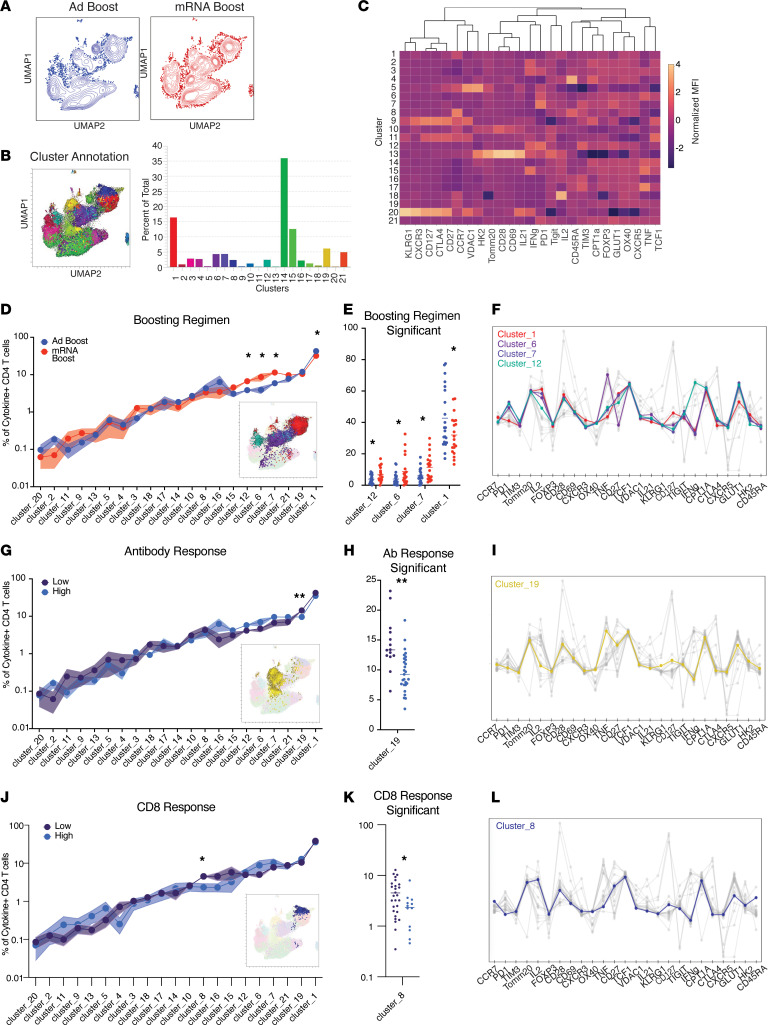
Homologous mRNA boost induces qualitatively different CD4^+^ T cells with increased metabolic response, cytokine production, and memory phenotype. (**A**) UMAP of total cytokine-producing (i.e., producing IL-2, TNF, IFN-γ, or IL-21) memory CD4^+^ T cells following overnight stimulation with BA.5 spike (S) peptides segregated by individuals receiving an adenoviral vector (Ad, blue, *n* = 21) boost compared with an mRNA boost (red, *n* = 20). (**B**) Xshift clustering algorithm detected 21 distinct clusters as shown on the UMAP and as a proportion of the entire S-specific T cell compartment. (**C**) Heatmap of normalized expression of all markers within flow cytometry panel according to Xshift cluster. (**D**) Frequency of clusters according to boosting regimen. Significance tested using repeated measures 2-way ANOVA with the Geisser-Greenhouse correction. (**E**) Individual values shown for significant clusters as determined in **D**. (**F**) Normalized expression of all markers with significant clusters highlighted. (**G**) Frequency of clusters according to individuals who mounted an Ab response above the positive cutoff (low *n* = 14, high *n* = 27). Significance tested using repeated measures 2-way ANOVA with the Geisser-Greenhouse correction. (**H**) Individual values shown for statistically significant clusters as determined in **G**. (**I**) Normalized expression of all markers with statistically significant clusters highlighted. (**J**) Frequency of clusters according to individuals who mounted CD8^+^ response above the average (low *n* = 28, high *n* = 13). Significance tested using repeated measures 2-way ANOVA with the Geisser-Greenhouse correction. (**K**) Individual values shown for statistically significant clusters as determined in **J**. (**L**) Normalized expression of all markers with statistically significant clusters highlighted. *P < 0.05, **P < 0.01. Data shown as mean ± SEM (in shaded bars for panels **D**, **G**, **J**) with each dot representing 1 individual.

In addition to cytokine production, we evaluated the phenotype of S-specific T cells using a high-dimensional (30-parameter) flow cytometry panel. This panel assesses expression of molecules indicative of T cell subsets, activation or exhaustion, and metabolic phenotypes (Supplemental Table 3). The ability of a T cell to engage differential metabolic programs upon activation, such as glycolysis, further defines a functional T cell in combination with the more commonly measured cytokine production. Cytokine-producing CD4^+^ memory T cells (i.e., producing IL-2, TNF, IFN-γ, or IL-21) following overnight stimulation with BA.5 peptides from a subset of 41 participants were first analyzed using uniform manifold approximation and projection (UMAP) as a data reduction approach (Figure 6A). Given the notable differences in phenotypes induced by mRNA or Ad vector boosting regimens (Figure 6A), we next used the unsupervised clustering algorithm, Xshift (52, 53), to define subclusters of S-specific T cells (Figure 6, B and C). Xshift identified a total of 21 clusters within the S-specific CD4^+^ compartment, which were then investigated for differential frequencies across various conditions (Figure 6, D–L).

There were no significant differences in S-specific CD4^+^ T cells induced by the ancestral or BA.5 peptides (Supplemental Figure 5A). Therefore, subsequent analysis focused exclusively on BA.5-specific responses. Three clusters of S-specific memory CD4^+^ T cells (clusters 12, 6, and 7) were expanded in recipients receiving an mRNA boost compared with an Ad boost (Figure 6, D and E). These clusters demonstrated cytokine production and increased activation, including upregulation of PD-1 and glucose transporter protein type 1 (GLUT1). GLUT1’s expression increases upon T cell activation to increase glucose uptake and support glycolysis (54, 55). Transient activation markers, such as CD69, are classically used in an antigen recall assay to assess recent activation by peptide stimulation. However, due to CD69 expression in a subset of peripheral T cells at baseline, antigen specificity is more accurately determined when accompanied by upregulation of additional activation markers, such as in the activation-induced marker assay or via cytokine production (56). We noted considerable CD69 expression in unstimulated conditions in SOTRs (Supplemental Figure 6, A and C), possibly due to ongoing immune activation resulting from alloimmune stimulation related to transplanted organ antigen recognition. In contrast, we found that GLUT1 was reliably upregulated upon peptide stimulation and may therefore represent a more specific marker of acute activation (Supplemental Figure 6B). Consistent with functional memory T cell responses, these 3 clusters also had elevated expression of costimulatory molecules CD27 and CD28 (Figure 6F and Supplemental Figure 5B). Generation of CD4^+^ T cells characterized by high CCR7 expression and low expression of activation markers PD-1 and GLUT1 (cluster 1) was less frequent in those boosted with mRNA compared with Ad vaccines (Figure 6F and Supplemental Figure 5B). In summary, we found that mRNA boosting was associated with S-specific CD4^+^ T cells with increased activation, cytokine production, and costimulatory molecule expression.

The quality of CD4^+^ T cell responses can directly impact the development of B cells, Ab titers, and CD8^+^ T cell responses. Therefore, to evaluate the correlation between phenotypes of S-specific CD4^+^ T cells and other immune responses, cluster frequencies were compared between patients who developed high or low Ab responses (defined as individuals with IgG titers above or below the positive manufacturer [MSD] threshold, respectively) and high or low CD8^+^ responses (defined as individuals who induced cytokine^+^ memory CD8^+^ T cells above or below the average frequency for SOTRs and healthy controls combined, respectively) (Figure 6, G–L). Cluster 19, characterized by cells with low GLUT1 expression that produced TNF alone, was significantly higher in patients with low Ab titers, suggesting insufficient CD4^+^ T cell help to support Ab production (Figure 6, H and I, and Supplemental Figure 5C). Low CD8^+^ responses were associated with increased frequency of cluster 8 (Figure 6, J and K), which demonstrates low levels of molecules shown to enhance T cell activation and/or proliferation. Cluster 8 produces IL-2 alone and expresses low GLUT1, VDAC, PD-1, and TCF1 (Figure 6L and Supplemental Figure 5D).

Together, these data demonstrate that mRNA and Ad boosting induce qualitatively different CD4^+^ T cells associated with S-specific Ab production and distinct CD8^+^ T cell phenotypes. In general, boosting with mRNA vaccines induced the highest percentage of IL-21^+^ pTfh cells and of activated CD4^+^ T cells, as determined by upregulation of PD-1, GLUT1, and costimulatory molecules following peptide stimulation. In contrast, boosting with an Ad vector generated CD4^+^ T cells with reduced polyfunctionality and low levels of GLUT1, indicating less functional CD4^+^ T cell responses that were correlated with poor Ab responses.

### CD8^+^ responses are not qualitatively different based on boosting regimen but are associated with differential Ab and CD4^+^ response.

**Figure 7:**
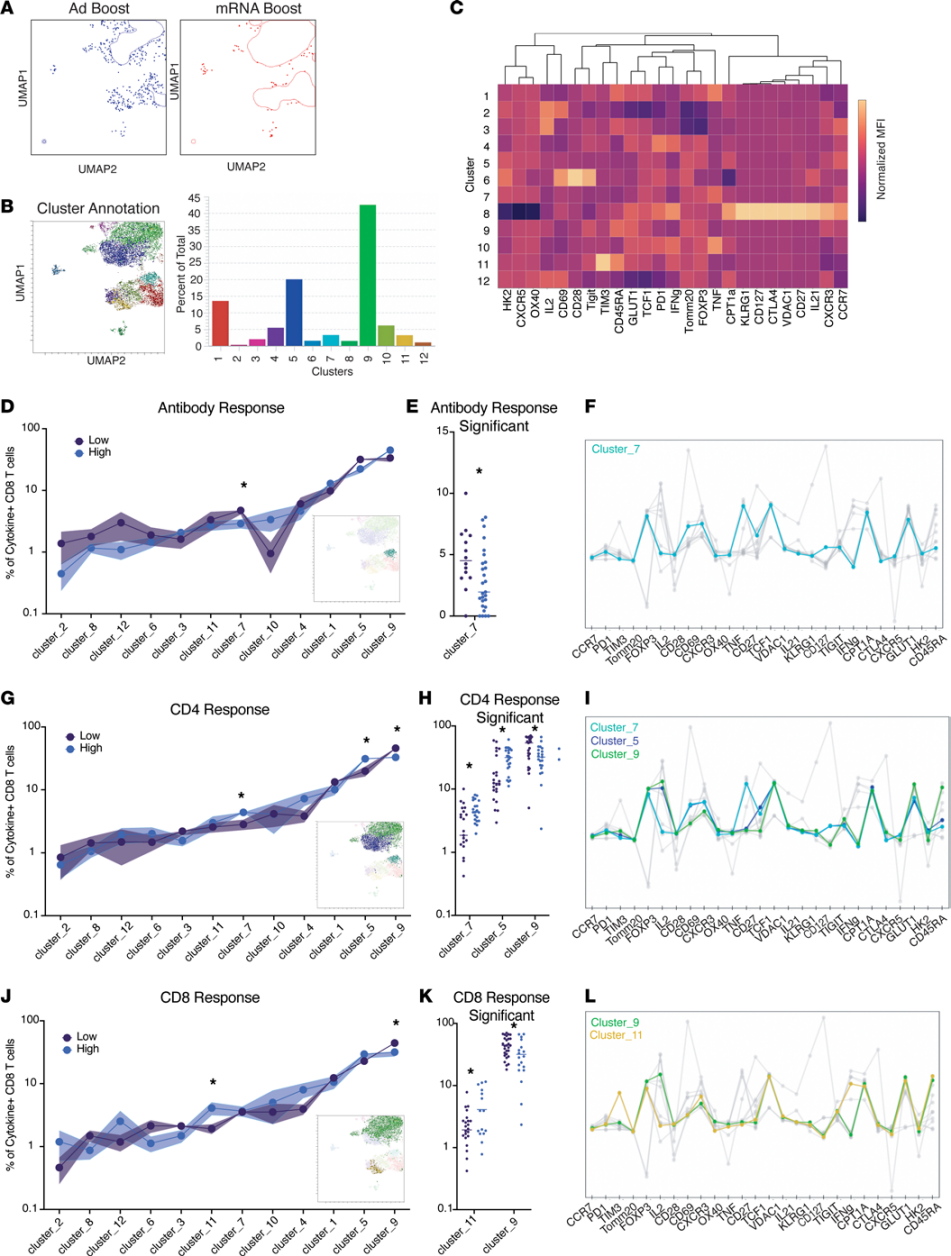
CD8^+^ responses are not qualitatively different based on boosting regimen but are associated with differential Ab and CD4^+^ response. (**A**) UMAP of total cytokine-producing (i.e., producing IL-2, TNF, IFN-γ, or IL-21) memory CD8^+^ T cells following overnight stimulation with BA.5 spike (S) peptides segregated by individuals receiving an adenoviral vector (Ad, blue, *n* = 21) boost compared with an mRNA boost (red, *n* = 20). (**B**) Xshift clustering algorithm detected 12 distinct clusters as shown on the UMAP and as a proportion of the entire S protein–specific compartment. (**C**) Heatmap of normalized expression of all markers within flow cytometry panel according to Xshift cluster. (**D**) Frequency of clusters according to individuals who mounted an Ab response above the positive cutoff (low *n* = 14, high *n* = 27). Significance tested using repeated measure 2-way ANOVA with the Geisser-Greenhouse correction. (**E**) Individual values shown for significant clusters as determined in **D**. (**F**) Normalized expression of all markers with significant clusters highlighted. (**G**) Frequency of clusters according to individuals who mounted a CD4^+^ response above the average (low *n* = 23, high *n* = 23). Significance tested using repeated measures 2-way ANOVA with the Geisser-Greenhouse correction. (**H**) Individual values shown for significant clusters as determined in **G**. (**I**) Normalized expression of all markers with significant clusters highlighted. (**J**) Frequency of clusters according to individuals who mounted a CD8^+^ response above the average (low *n* = 28, high *n* = 13). Significance tested using repeated measures 2-way ANOVA with the Geisser-Greenhouse correction. (**K**) Individual values shown for significant clusters as determined in **J**. (**L**) Normalized expression of all markers with significant clusters highlighted. *P < 0.05, **P < 0.01, ***P < 0.001, and ****P < 0.0001. Data shown as mean ± SEM (in shaded bars for panels **D**, **G**, **J**) with each dot representing 1 individual.

We next investigated the phenotype of BA.5 S-specific CD8^+^ T cells using UMAPs and Xshift clustering, as performed for CD4^+^ responses (Figure 7, A–C). In line with similar frequencies of CD8^+^ T cells induced by both heterologous and homologous boosting, there were no significant differences in clusters between individuals receiving the mRNA or Ad boosting regimen (Figure 7A and Supplemental Figure 7A). Unlike in the CD4^+^ compartment, there were no qualitative differences in the T cells induced by the different vaccine regimens. When participants were stratified by ability to mount a high Ab, CD4^+^, or CD8^+^ response, there were differentially expressed clusters within the S-specific CD8^+^ compartment (Figure 7, D–L). Participants with lower Ab titers had a higher frequency in CD8^+^ T cells of cluster 7, which, like cluster 19 in the CD4^+^ compartment, had low GLUT1 expression and produced a single cytokine, TNF (Figure 7, D–F, and Supplemental Figure 7B). Both clusters were associated with a poor Ab response. However, cluster 7 in the CD8^+^ compartment was associated with increased overall CD4^+^ T cell responses (Figure 7, G–I, and Supplemental Figure 7C). While this may seem contrary to cluster 7 correlating with a low Ab response, the total CD4^+^ response represents the ability to make any cytokine and cluster 7 correlated with CD4^+^ T cells making TNF, not IL-21. CD4^+^ T cells producing IL-21 were highly correlated with increased Ab titers (Figure 2). Given that cluster 7 most strongly correlated with TNF^+^CD4^+^ responses and poor Ab responses, individuals who preferentially mount a TNF-skewed response in both the CD4^+^ and CD8^+^ compartment and not IL-21 may be less likely to generate high Ab responses (Supplemental Figure 7D). Low CD4^+^ responses were also associated with a CD45RA^+^ TEMRA population (cluster 9) that produced IL-2. Finally, individuals with higher CD8^+^ responses demonstrated increased cluster 11 that was dominated by IFN-γ production (Figure 7, J–L, and Supplemental Figure 7E). These results indicate that IL-2 and TNF may be more readily produced by CD8^+^ T cells in this patient population and that IFN-γ production is restricted to those with above-average CD8^+^ responses. These data also suggest that IFN-γ is likely a better readout for true antigen specificity in the CD8^+^ compartment in SOTRs.

## Discussion

Given impaired Ab neutralization of currently circulating VOC, CD8^+^ T cell responses to vaccination might serve as a critical second line of defense (57). The need for CD8^+^ T cell responses may be amplified in SOTRs, who demonstrate not only lower Ab titers but also reduced neutralizing capacity following 2- and 3-dose vaccination when compared with healthy individuals. To identify the vaccine regimens that induced strain–cross-reactive T cell responses in SOTRs, we evaluated S-specific CD4^+^ and CD8^+^ T cell responses in SOTRs following a third dose of homologous mRNA or heterologous Ad vector SARS-CoV-2 vaccination, as well as anti-S IgG titers and pseudo-neutralization. Cellular immunity to earlier Omicron VOC has been shown to be largely maintained in healthy individuals despite the mutations in the S protein (58–61), but whether cross-reactive T cell responses to BA.5 are induced by vaccination has not yet been comprehensively evaluated in SOTRs. While homologous boosting increased anti-S IgG titers compared with heterologous boosting, pseudo-neutralization remained significantly reduced against BA.5 compared with the ancestral strain (*P* < 0.0001). In contrast to Abs, T cells retained comparable reactivity against BA.5, consistent with previous reports in healthy individuals and earlier reports of BA.1–cross-reactive T cells in SOTRs (62). Within this cohort of SOTRs, homologous mRNA boosting induced significantly higher CD4^+^ responses, with mRNA-1273 preferentially inducing polyfunctional/IL-21^+^CD4^+^ T cells that correlated with higher Ab titers. Neither homologous mRNA nor heterologous Ad vector boosting generated robust CD8^+^ T cell responses. These data demonstrate that boosting with the ancestral strain can induce cross-reactive T cell responses against emerging VOCs; however, alternative vaccination strategies are still required to induce robust CD8^+^ responses in SOTRs.

Several lines of evidence point toward the importance of T cell responses, in addition to Ab responses, in protection against SARS-CoV-2 infection. SARS-CoV-2 challenge models in rhesus macaques demonstrate that a lack of protection was associated with both low CD8^+^ responses and low- to mid-level Ab titers (63, 64). Animals with low Ab titers that mounted a CD8^+^ response were protected from severe infection despite poor Ab responses, demonstrating a role for CD8^+^ responses when Ab titers are low. Consistent with these results, BNT162b2 demonstrated 70% efficacy against severe BA.1 infection despite a loss of Ab neutralization (65). These results indicate that T cell responses and/or non-neutralizing function of Abs may provide additional vaccine-mediated protection. Preclinical data from vaccines against other pathogens demonstrate potent induction of CD8^+^ T cell responses with Ad-based vaccines and suggest potential to preferentially enhance T cell responses with the addition of an Ad boost. In contrast, we detected CD4^+^ responses following all SARS-CoV-2 vaccine regimens, yet neither regimen induced robust CD8^+^ T cell responses compared with vaccines against other pathogens (66–69). Consistent with our findings, a recent study that randomized kidney transplant recipients to either Ad vector boost or mRNA boost found no significant difference in Ab responses or bulk T cell responses as measured by ELISPOT (70). To expand upon these previously generated data, our study provides important information regarding the relative contributions of the CD4^+^ and CD8^+^ T cell compartments.

In contrast to CD8^+^ responses, we found that homologous boosting with mRNA vaccines induced higher levels of polyfunctional CD4^+^ T cells in response to BA.5 compared with an Ad boost. While CD4^+^ T cells produced combinations of Th1 cytokines (IL-2, IFN-γ, and TNF), Tfh CD4^+^ cells producing IL-21 alone or as part of a polyfunctional response correlated best with Ab titers. These data are consistent with reports demonstrating the importance of Tfh cells in driving B cell development and maturation within the germinal center and a correlation between pTfh cells and responses to multiple vaccines in healthy individuals and to SARS-CoV-2 vaccines in SOTRs (6, 43, 71–74). Our data suggest that mRNA vaccines’ ability to specifically enhance Tfh cell responses may partially explain their capacity to increase Ab titers compared with other platforms. Although B cell responses were not evaluated in this study, increased Tfh cells have been shown to provide enhanced T cell help to B cells and promote increased Ab titers (75). Despite small sample sizes in our study, 3 doses of mRNA-1273 induced significantly higher levels of polyfunctional CD4^+^ T cells including IL-21^+^ pTfh cells and anti-S IgG titers compared with all other vaccine regimens evaluated. Although the 2 authorized mRNA vaccines are similar, they have demonstrated differential induction of Ab titers, neutralization, and non-neutralizing Ab function in healthy individuals (41, 76). These differences could be mediated by factors such as different antigen doses, intervals between initial doses, or differing composition of the lipid nanoparticle. Future work will be needed to uncover the roles each of these variables play in driving CD4^+^ T cell responses; however, differences between the 2 mRNA platforms in inducing polyfunctional CD4^+^ T cell or Tfh cell activation could help explain increased titers following vaccination with the mRNA-1273 vaccine.

Our data demonstrate that alternative vaccination approaches and/or alternative means of protection are still required for SOTRs, given the lack of conventional CD8^+^ T cell responses following the investigated vaccine regimens. Although CD8^+^ T cells are often considered the primary cytotoxic lymphocyte induced by vaccination, vaccination with either mRNA-1273 or BNT162b2 induced a substantial proportion of CD4^+^ cytotoxic T lymphocytes (CD4-CTLs) in healthy individuals, as assessed by induction of granzyme B^+^ and intracellular CD40L (41). Future work should evaluate the presence of CD4-CTLs in SOTRs as an additional means to promote protection. Nevertheless, increasing CD8^+^ T cell responses in addition to CD4-CTLs remains a goal of vaccine strategies. To achieve this goal, optimizing the timing and order of vaccination could improve the induction of CD8^+^ T cell responses. Accordingly, in a recent study evaluating responses to SARS-CoV-2 vaccination in individuals with cancer, heterologous boosting was shown to significantly increase CD8^+^ T cell responses (77). However, participants in the described study received 2 doses of ChAdOx1 as a primary series followed by a third dose of BNT162b2. Therefore, priming with an Ad vector or utilizing alternative Ad-based vaccines, such as those using ChAdOx1, may help improve CD8^+^ responses in SOTRs.

Finally, unveiling the metabolic circuits regulating immunity following vaccination may provide additional means to enhance induction of durable, cross-reactive T cell memory responses, key to improving vaccine development. Work to delineate metabolic mechanisms dictating vaccine response has only begun and has largely focused on healthy individuals (78–82). Our data suggest that GLUT1 may be a better marker of recent T cell activation than CD69 and, when combined with PD-1, expression can differentiate antigen-specific T cells associated with improved responses. This may be particularly relevant in immunosuppressed individuals, where immunosuppressive drug regimens may prevent full activation of T cells (83). For example, we found that a subset of individuals generated both CD4^+^ and CD8^+^ T cell responses skewed toward TNF production. While the presence of these cells indicates a detectable T cell response induced by vaccination, we found that they were associated with low T cell metabolic activity and lower Ab responses. Therefore, combining markers of metabolic activity with assessment of cytokine production can provide a more nuanced understanding of the quality and nature of cellular responses. Although we investigated T cell responses using a panel designed to assess T cell exhaustion, no significant differences in combined expression of coinhibitory molecules (TIGIT, CTLA4, TIM3, PD-1) were observed based on vaccine regimen or vaccine response. Therefore, prototypical signs of T cell exhaustion may not be the determining factor driving a lack of response in immunosuppressed individuals. Instead, we propose that analysis of engagement of glycolysis and upregulation of glycolytic machinery in T cells may provide more insight into functional vaccine responses in SOTRs.

There are certain limitations to the work. One main limitation of this work is that the threshold for protective T cell responses in COVID-19 has not been defined, either in healthy controls or in SOTRs. However, generating comprehensive data on the frequency and phenotype of vaccine-elicited T cell responses will be critical to correlate with future breakthrough infection outcomes to begin to define this threshold. We did not perform live virus neutralization assays on these samples, the current gold standard for Ab neutralization, or evaluate antigen-specific B cells to improve our understanding of the vaccine-induced Ab response. Instead, we utilized a surrogate neutralization assay of ACE2 inhibition, which we have demonstrated previously has a high degree of correlation with subvariant live virus neutralization activity (5, 42). Future work will evaluate S-specific B cells and the correlation with T cell responses in this cohort. Finally, we were limited by the availability of samples within our observational cohort from individuals who were primed with an Ad vector to investigate how alternative priming strategies could influence responses. Given the earlier emergency use authorization for mRNA COVID-19 vaccines than JNJ-78436735 in the United States, our study assessed priming with either mRNA-1273 or BNT162b2, the most common prime used in the United States. Thus, we could not assess whether multiple doses of JNJ-78436735 or ChAdOx1 or alternative dosing schedules could have increased CD8^+^ T cell responses. Despite these limitations, our work substantially contributes to the understanding of the development of T cell responses following vaccination in SOTRs.

Together, our data demonstrate that, even in immunosuppressed individuals, BA.5-specific T cell responses are induced by vaccination, with evidence of both quantitative and qualitative differences induced by homologous mRNA versus heterologous Ad vector boosting. Whether bivalent boosting inclusive of Omicron S sequences enhances BA.5-specific T cell frequency and function in SOTRs remains unknown. However, our study demonstrates that ancestral strain vaccination does induce cross-reactive T cell responses and antigenic updates may not be required to generate VOC-reactive T cell responses. Our data also highlight correlations between specific CD4^+^ T cell subsets and Ab induction, permitting future studies that assess whether bivalent vaccination induces CD4^+^ T cell responses that support de novo BA.5-specific Ab responses in immunosuppressed individuals. In summary, we demonstrate that mRNA and Ad boosting induce distinct Ab and T cell responses, which in conjunction with assessment of infection outcome, lay the foundation to select vaccination strategies that induce optimal Ab and T cell responses.

## Methods

### PBMC preparation.

Blood was collected in acid citrate dextrose or heparin tubes, and plasma was isolated by centrifugation and stored at –80°C until Ab titers were measured. PBMCs were isolated within 24 hours of blood collection, as previously described (84). Aliquots of PBMCs were stored in liquid nitrogen until further analysis.

### Ab titers.

Plasma was thawed and anti-N, anti-RBD, and anti-S IgG were measured using the multiplex chemiluminescent MSD V-PLEX COVID-19 Respiratory Panel 3 Kit according to the manufacturer’s protocol at a dilution of 1:5,000. Plates were read on MSD QuickPlex SQ 120, and arbitrary units were calculated using MSD Discovery Workbench software according to the manufacturer’s protocol. Conversion to WHO binding antibody units per milliliter was done by multiplying by the manufacturer’s recommended conversion factor. The MSD assays were performed according to the manufacturer’s instructions, including the recommended sample dilutions. The positivity cutoff was determined by the manufacturer based on pre-pandemic serum samples and PCR-confirmed cases during the pre–SARS-CoV-2 vaccine period of the pandemic.

### ACE2 inhibition assay.

The MSD ACE2 inhibition assay was used to measure the inhibition of ACE2 receptor binding to the S protein (% ACE2 inhibition) as previously described (5). All samples were assayed on MSD V-PLEX SARS-CoV-2 panel 27 at a dilution of 1:100. Plates were read on MSD QuickPlex SQ 120, and percentage inhibition was calculated using the manufacturer’s protocol.

### Antigen recall assay.

For assessment of S-specific cytokine production, PBMCs were restimulated in vitro. PBMCs were thawed using a CryoThaw adaptor (85) (Medax) into 10 mL of prewarmed RPMI (Gibco) supplemented with 10% FBS (Atlanta Biologicals). Samples were rested for approximately 6 hours following thaw. Following the rest, 1.5 × 10^6^ cells were cultured in 200 μL of RPMI supplemented with 10% FBS per stimulation in a 96-well, round-bottom plate. Samples were stimulated with 1 μg/mL ancestral or BA.5 SARS-CoV-2 S peptides that had been resuspended in DMSO (Peptides and Elephants) in the presence of 10 μg/mL brefeldin A (MilliporeSigma) overnight (~16 hours). Unstimulated wells were supplemented with equivalent volume DMSO and brefeldin A for all samples. Following overnight stimulation PBMCs were stained for flow cytometry. Samples were run in 2 batches with participants from each of the 4 vaccine regimens described above distributed evenly between the 2 batches. All samples had individual unstimulated conditions (DMSO only) and stimulated conditions (ancestral or BA.5) and were all background subtracted. If a sample was negative or 0, it was changed to the lowest detectable value on the day the samples were run. These were considered nonresponse. Peptides were prescreened for background activity in pre-pandemic samples and determined to have background activity comparable to DMSO-only controls.

### Flow cytometry staining.

Flow cytometry Abs used for phenotypic and metabolic analyses can be found in Supplemental Table 3. Cells were washed once in PBS and immediately stained for viability with BioLegend Live/Dead Zombie NIR Fixable Viability Dye and BD Fc Block for 10 minutes at room temperature. Cell surface staining was performed in 100 μL of 20% BD Horizon Brilliant Stain Buffer + PBS with surface stain Ab cocktail for 20 minutes at room temperature. Cells were fixed and permeabilized with eBioscience FoxP3/Transcription Factor Staining Buffer Set with 1× Fixation/Permeabilization reagent for 20 minutes at room temperature. Cells were washed with 1× Permeabilization/Wash buffer. Intracellular staining (ICS) was performed in 100 μL 1× Permeabilization/Wash buffer with ICS Ab cocktail for 20 minutes at room temperature. Cells were washed once with Permeabilization/Wash buffer, then resuspended in 1% paraformaldehyde for acquisition by flow. Samples were run on a 4-laser Cytek Aurora spectral flow cytometer.

### Flow cytometry analysis.

FCS files were analyzed using FlowJo v10 (10.6.2) software using manual gating and plugins for UMAP and Xshift. Frequencies of clusters identified by Xshift were applied to individual samples, and frequency of total population was determined. These frequencies were then stratified according to indicated parameter and analyzed in GraphPad Prism 8. Polyfunctional T cell responses were analyzed using Pestle v2.0 and SPICE v6.0(86). To avoid any confounding issues associated with batch effect on mean fluorescence intensity, high-dimensional analysis as performed in Figures 5 and 6 was only performed on samples run in the same batch. Batches were designed to evenly distribute participants according to vaccine regimen.

### Statistics.

Statistical calculations were performed in GraphPad Prism 8 or SPICE v6. Multivariate correlation was performed using JMP Pro v16 by calculating Spearman’s ρ coefficients within the multivariate platform. Data are shown as mean ± SEM unless otherwise noted. Specific statistical tests are noted in figure legends. A 2-sided *P* value less than 0.05 was considered significant. Multivariable linear regression was utilized to assess the association of vaccine series (primary exposure: mRNA versus Ad vector) with spike-specific CD4^+^ and CD8^+^ T cell cytokine expression (primary outcomes: overall and IL-21), after adjusting for key clinical and transplant factors previously shown to associate with vaccine response in SOTRs. Covariables included recipient age at vaccination (≥65 years versus <65 years), time since transplant (≥5 years versus <5 years), transplant type (liver-only versus other organ), antimetabolite therapy (any versus none), and belatacept use (any versus none). Collinearity was assessed via variance inflation factor. Crude and adjusted β coefficient values for the primary exposure variable in each model are presented in Supplemental Table 2.

### Study approval.

SOTRs were enrolled in a national, prospective, observational cohort, “COVID-19 antibody testing of recipients of solid organ transplants and patients with chronic diseases,” which was approved by the Johns Hopkins IRB (no. 00248540), as previously described (5, 9, 10). All SOTRs were recruited virtually and provided detailed transplant history as well as oral informed consent (waiver of written consent granted by the IRB). All vaccines were also administered independently in the community at the discretion of participants and their transplant providers. The cohort consisted of participants who did not have a substantial Ab response after the 2-dose mRNA series. For analysis, participants were selected based on adequate sample availability approximately 2 weeks following the third dose. We selected individuals who fit into 1 of 4 categories: 3 doses of mRNA-1273, 2 doses of mRNA-1237 followed by JNJ-78436735, 3 doses of BNT162b2, or 2 doses of BNT162b2 followed by JNJ-78436735. All participants within the observational cohort that fit these criteria and had sufficient biobanked PBMCs were evaluated for Ab titers and T cell responses (Table 1). All participants with prior history of SARS-CoV-2 or detectable anti-nucleocapsid Abs indicative of prior infection were excluded from the analysis. All healthy control samples were collected at Emory University. Collection and processing were performed under approval from the university IRB (no. 00002061). Adults ≥ 18 years were enrolled who met eligibility criteria and provided informed consent. Participants submitted an enrollment form containing a waiver of documentation of consent. Submitting the enrollment form constituted as consent to enrolling in the study. All patient samples were deidentified prior to processing and testing.

## Author contributions

EAT, WAW, AHK, JNB, and ALC conceived the study. EAT, KC, and AHK developed methodology. EAT, WN, LS, TSJ, and AC investigated. EAT and ALC visualized data. WAW, DLS, and ALC acquired funding. ATA, JLA, AART, WAW, DLS, JNB, and ALC were project administrators. JNB and ALC supervised. EAT and ALC wrote the original draft. EAT, WN, LS, TSJ, AC, KC, JLA, ATA, HS, NR, AART, DLS, WAW, AHK, JNB, and ALC reviewed and edited the manuscript.

## Supplementary Material

Supplemental data

ICMJE disclosure forms

## Figures and Tables

**Table 1:**
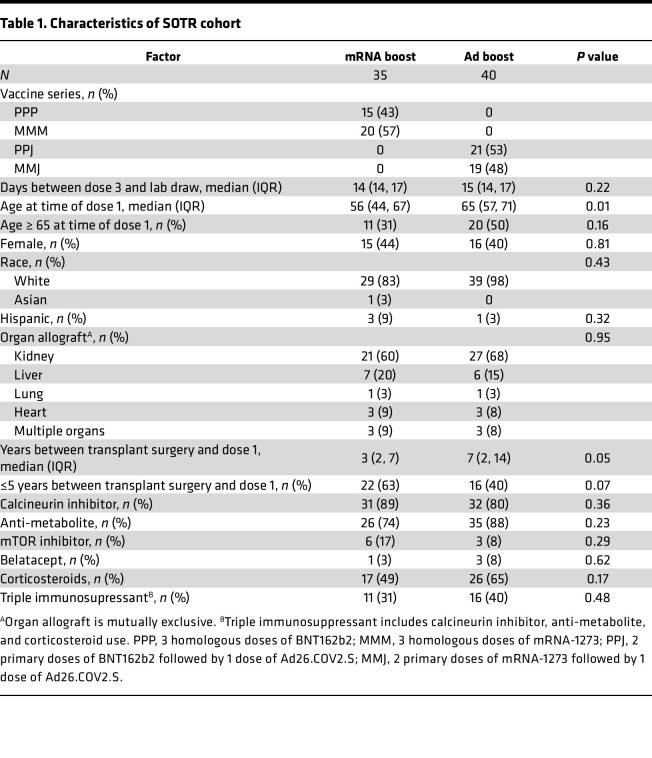
Characteristics of SOTR cohort

## Version 1 04/27/2023

In-Press Preview

## Version 2 05/22/2023

Electronic publication
