# Supplementary material for: Heterologous versus homologous boosting elicits qualitatively distinct, BA.5–cross-reactive T cells in transplant recipients
Source: JCI Insight. 2023 May 22;8(10):e168470. doi: 10.1172/jci.insight.168470 (PMC10322695; doi:10.1172/jci.insight.168470)
Supplement: Supplemental data [file jciinsight-8-168470-s052.pdf]

Thompson EA, et al: Heterologous versus homologous boosting regimens elicit qualitatively distinct, BA.5-cross reactive T cells in transplant recipients.

Supplementary Figures

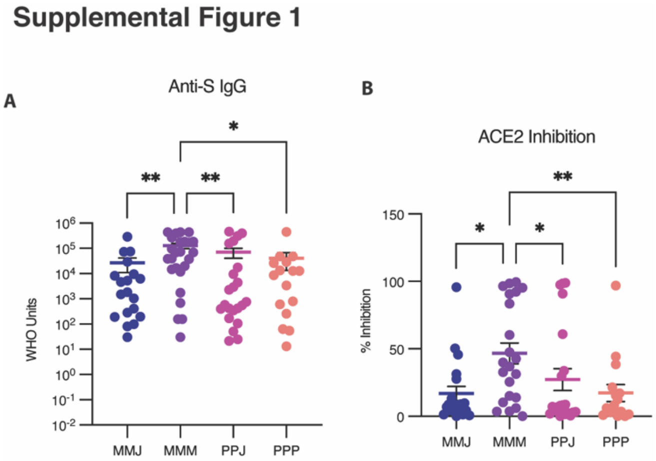

Figure S1: Anti-Spike titers and ACE2 inhibition by vaccine regimen

A. Anti-spike (S) IgG titers as determined by MSD research assay in participants that received two doses of Moderna mRNA mRNA-1273 followed by Johnson and Johnson adenoviral vector JNJ-78436735 (MMJ; n=19), three doses of Moderna mRNA-1273 (MMM; n=20), two doses of Pfizer BNT162b2 followed by Johnson and Johnson JNJ-78436735 (PPJ; n=21), or three doses of Pfizer BNT162b2 (PPP; n=15). Samples were studied approximately 2 weeks following the third dose. Significance tested using Kruskal-Wallis test. **B**. Ancestral ACE2 inhibition. Significance tested using Kruskall-Wallis test. *p < 0.05, **p < 0.01, ***p < 0.001, and ****p < 0.0001. All data shown as Mean±SEM with each dot representing an individual.

Supplemental Figure 2

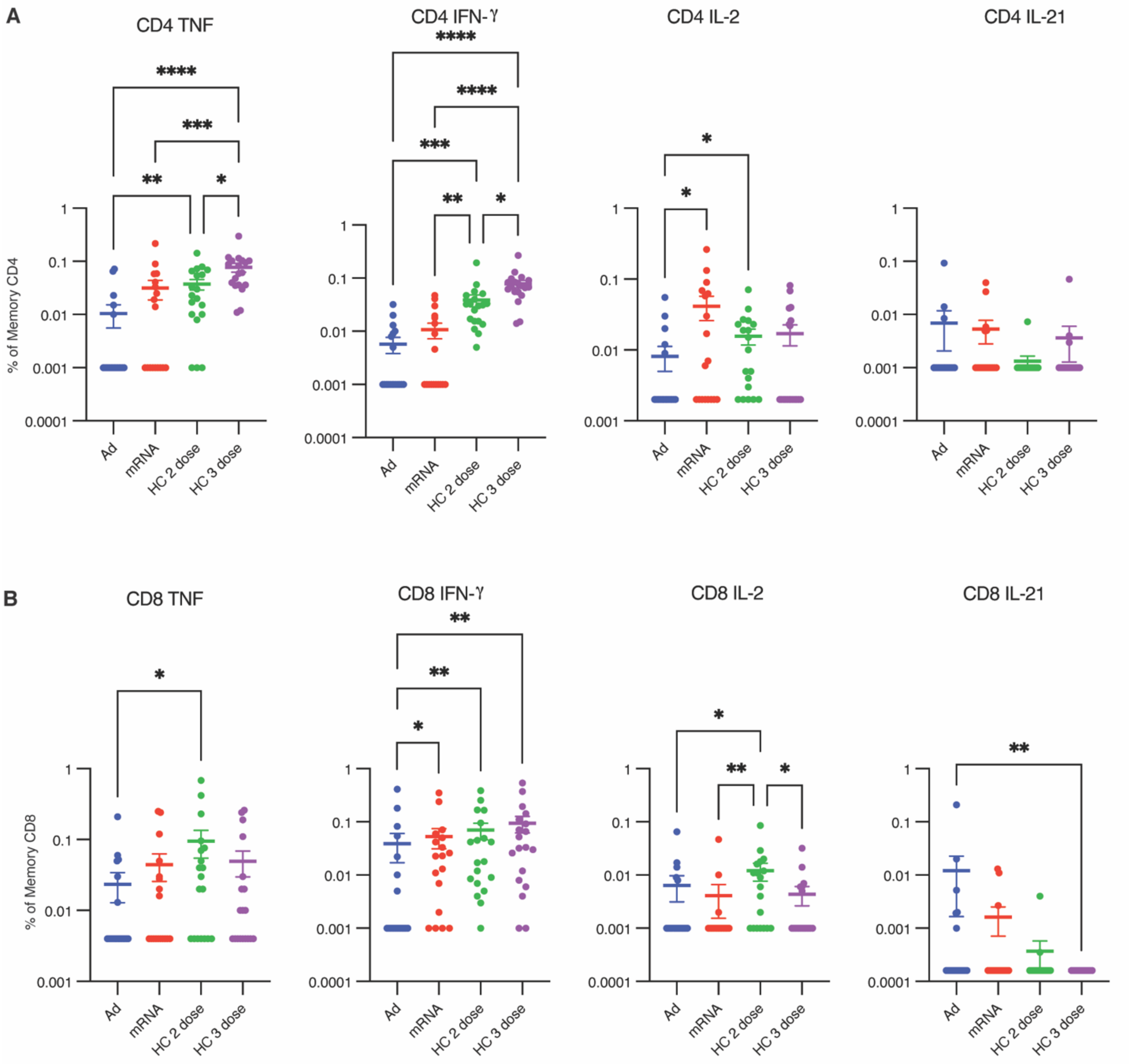

Figure S2: T cell responses compared to healthy controls

Peripheral blood mononuclear cells (PBMCs) were stimulated overnight with overlapping peptides (15mers overlapping by 11) against ancestral (WA1) spike (S) protein. Spike-specific T cell responses were evaluated in SOTRs that received two doses of an mRNA COVID-19 vaccine followed by Adenoviral vector boost (Ad Boost, Blue, n=20) or a third mRNA dose (mRNA Boost, red, n=18). Samples were collected approximately 2 weeks following the third dose. Healthy controls received two doses (HC 2 dose, n=19) or three doses of BNT162b2 (HC 3 dose, n=19). A-B. Frequency of memory CD4 (A) or CD8 (B) T cells producing TNF, IFN-γ, IL-2, or IL-21. All values are with unstimulated DMSO-only control levels subtracted. Samples with negative or zero values were converted to the lowest detected value for visualization purposes. Significance tested using Kruskal-Wallis test. *p < 0.05, **p < 0.01, ***p < 0.001, ****p < 0.0001, and ns= non-significant. All data shown as Mean±SEM with each dot representing an individual.

Supplemental Figure 3

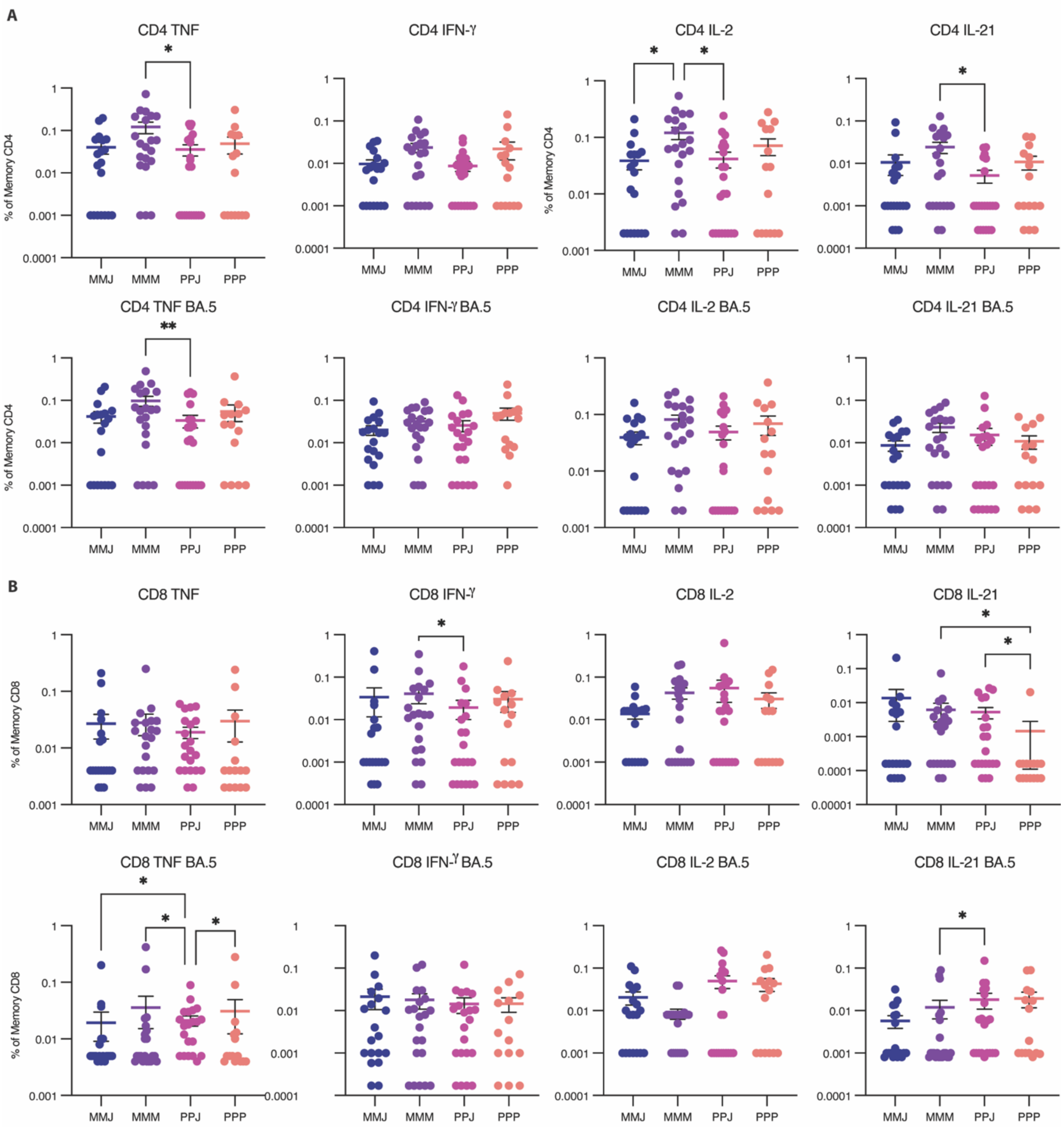

Figure S3: T cell responses by vaccine regimen

Peripheral blood mononuclear cells (PBMCs) were stimulated overnight with overlapping peptides (15mers overlapping by 11) against ancestral (WA1) or BA.5 spike (S) protein. Participants received two doses of Moderna mRNA mRNA-1273 followed by Johnson and Johnson adenoviral vector JNJ-78436735 (MMJ; n=19), three doses of Moderna mRNA-1273 (MMM; n=20), two doses of Pfizer BNT162b2 followed by Johnson and Johnson JNJ-78436735 (PPJ; n=21), or three doses of Pfizer BNT162b2 (PPP; n=15). A-B. Frequency of memory CD4 T cells (A) or memory CD8 T cells (B) producing individual cytokine as noted. Significance tested using Kruskal-Wallis test. *p < 0.05, **p < 0.01, ***p < 0.001, and ****p < 0.0001. All data shown as Mean±SEM with each dot representing an individual.

Supplemental Figure 4

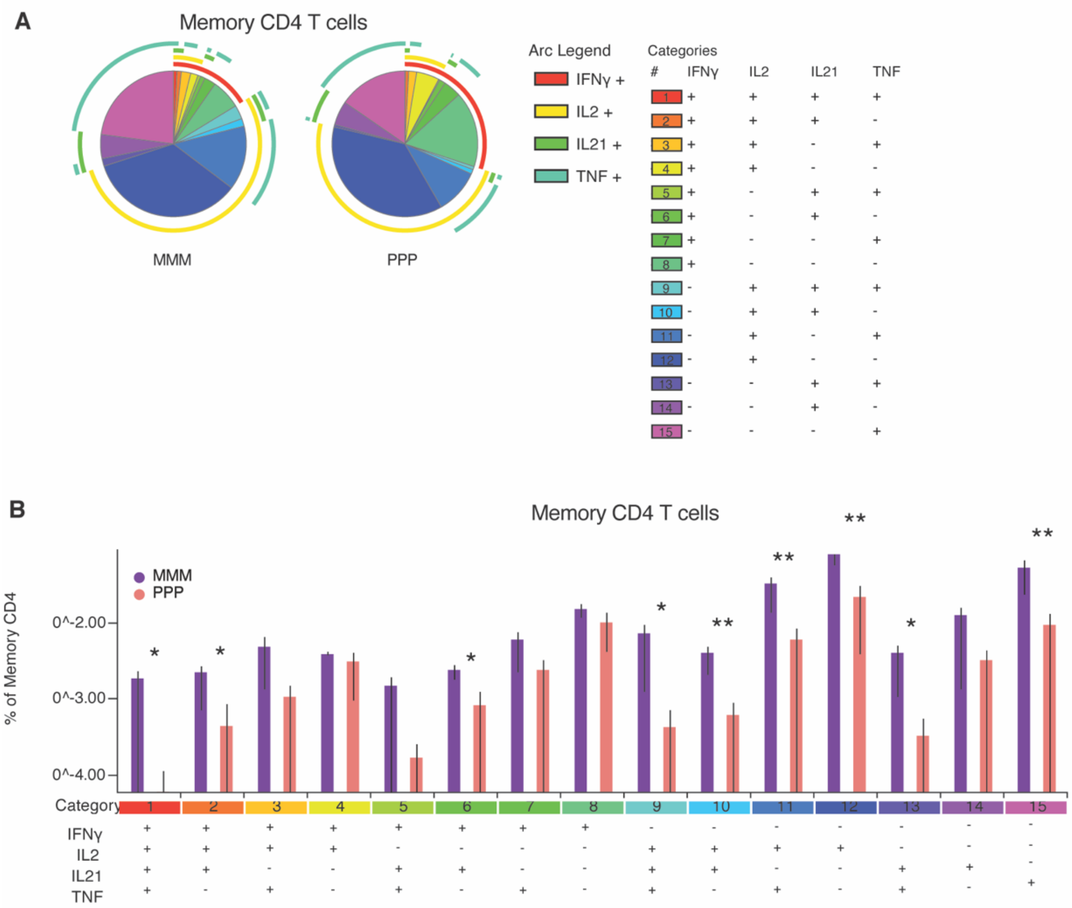

***Figure S4**: Increased polyfunctional CD4 T cells following three doses of mRNA-1273 compared to BNT162b2*

Spike-specific T cell responses were evaluated in participants that received three doses of Moderna mRNA-1273 (MMM; n=20) or three doses of Pfizer BNT162b2 (PPP; n=15). Polyfunctionality of the memory CD4 T cell responses. Pie charts show the fraction of total cytokine response comprising any combination of IFNγ, IL-2, TNF, or IL-21. Pie arcs show the proportion making each cytokine as annotated. **A.** Comparison of CD4 response against BA.5 peptides, segregated by boosting regimen. **B.** Overview of CD4 response against BA.5 peptides, with percent of total memory CD4 T cells shown for individual polyfunctional categories. Significance tested using the Wilcoxon rank sum test as calculated in SPICE v6. *p < 0.05, **p < 0.01, ***p < 0.001, and ****p < 0.0001. All data shown as Mean±SEM.

Supplemental Figure 5

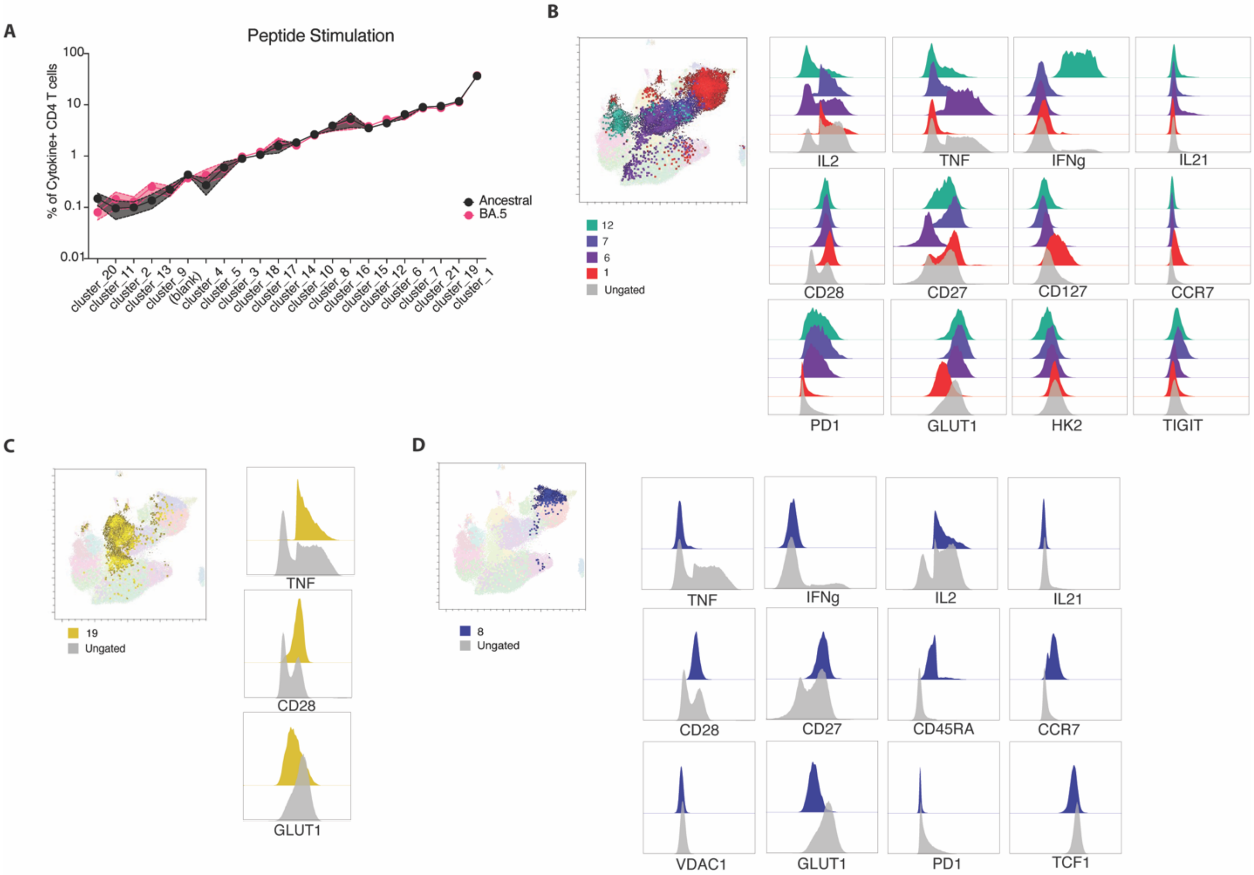

Figure S5: Phenotype of significant clusters identified in CD4 T cell compartment
UMAP projection of total cytokine-producing (i.e. producing IL-2, TNF, IFN-γ, or IL-21) memory CD4 T cells following overnight stimulation with BA.5 spike (S) peptides with Xshift clusters identified as significant highlighted. Histograms show markers that were most differentially expressed by indicated clusters. **A**. Frequency of clusters according to stimulating peptide pool. Significance tested using repeated measures two-way ANOVA with the Geisser-Greenhouse correction. No significance detected **B.** Significant clusters according to boosting regimen. **C**. Significant clusters according to individuals that mounted an antibody (Ab) response above the positive cutoff. **D**. Significant clusters according to individuals that mounted CD8 response above the average.

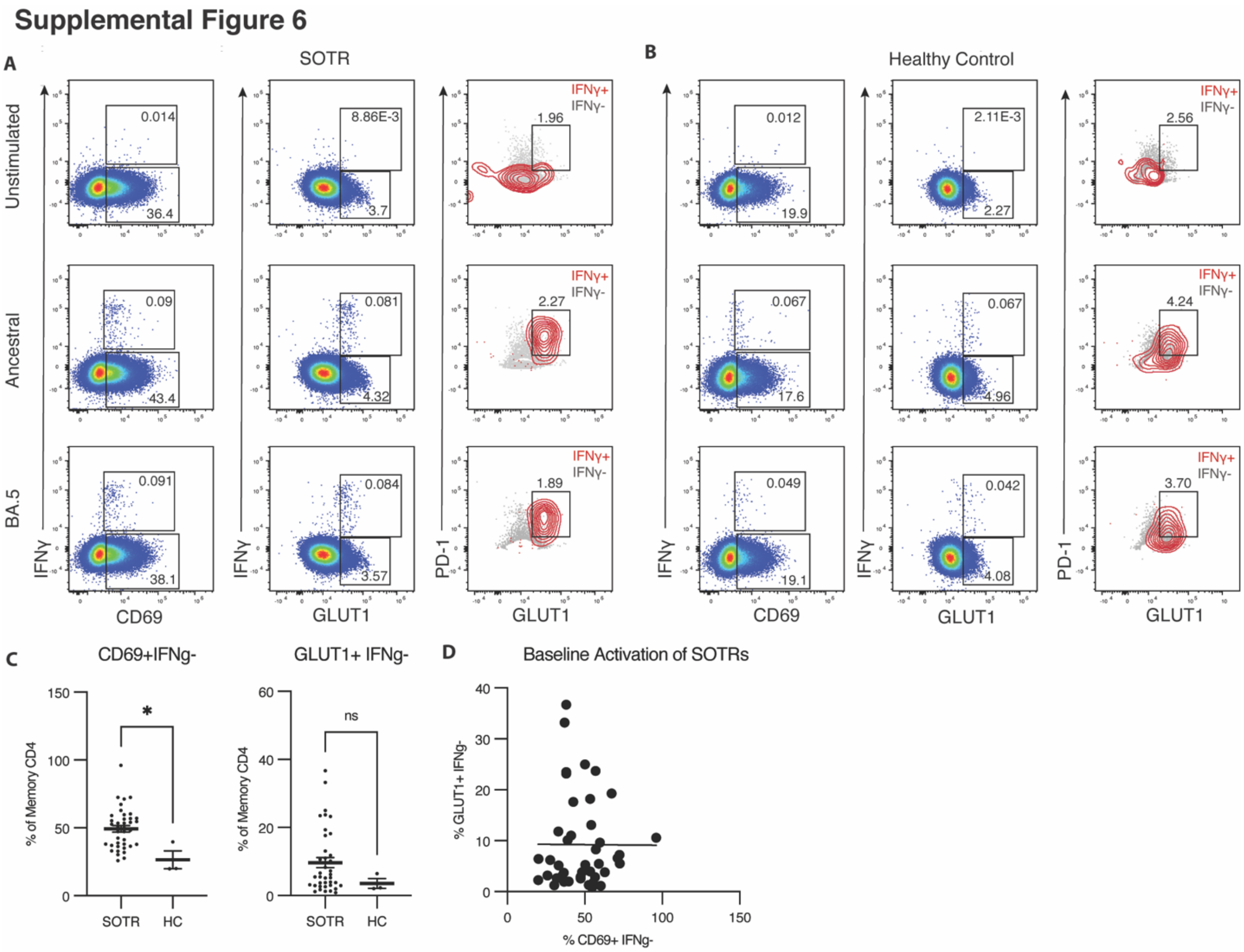

Figure S6: Baseline activation in SOTRs

Peripheral blood mononuclear cells (PBMCs) were stimulated overnight with overlapping peptides (15mers overlapping by 11) against ancestral (WA1) or BA.5 spike (S) protein. A-B. Representative gating of memory CD4 T cells in SOTR (A) and healthy controls (B). SOTRs reliably upregulate GLUT1 upon activation and IFN-γ+ CD4 T cells are GLUT1+ and PD-1+ in SOTRs. C. Frequency of CD69+ IFN-γ- or GLUT1+ IFN-γ- cells out of total memory CD4 compartment in unstimulated conditions. Significance tested using Mann-Whitney U test. D. Correlation of GLUT1+ IFN-γ- CD4 T cells with CD69+ IFN-γ- T cells demonstrates that increased baseline CD69 expression in SOTRs does not correlate with increased GLUT1 expression. *p < 0.05, **p < 0.01, ***p < 0.001, and ****p < 0.0001. All data shown as Mean±SEM.

Supplemental Figure 7

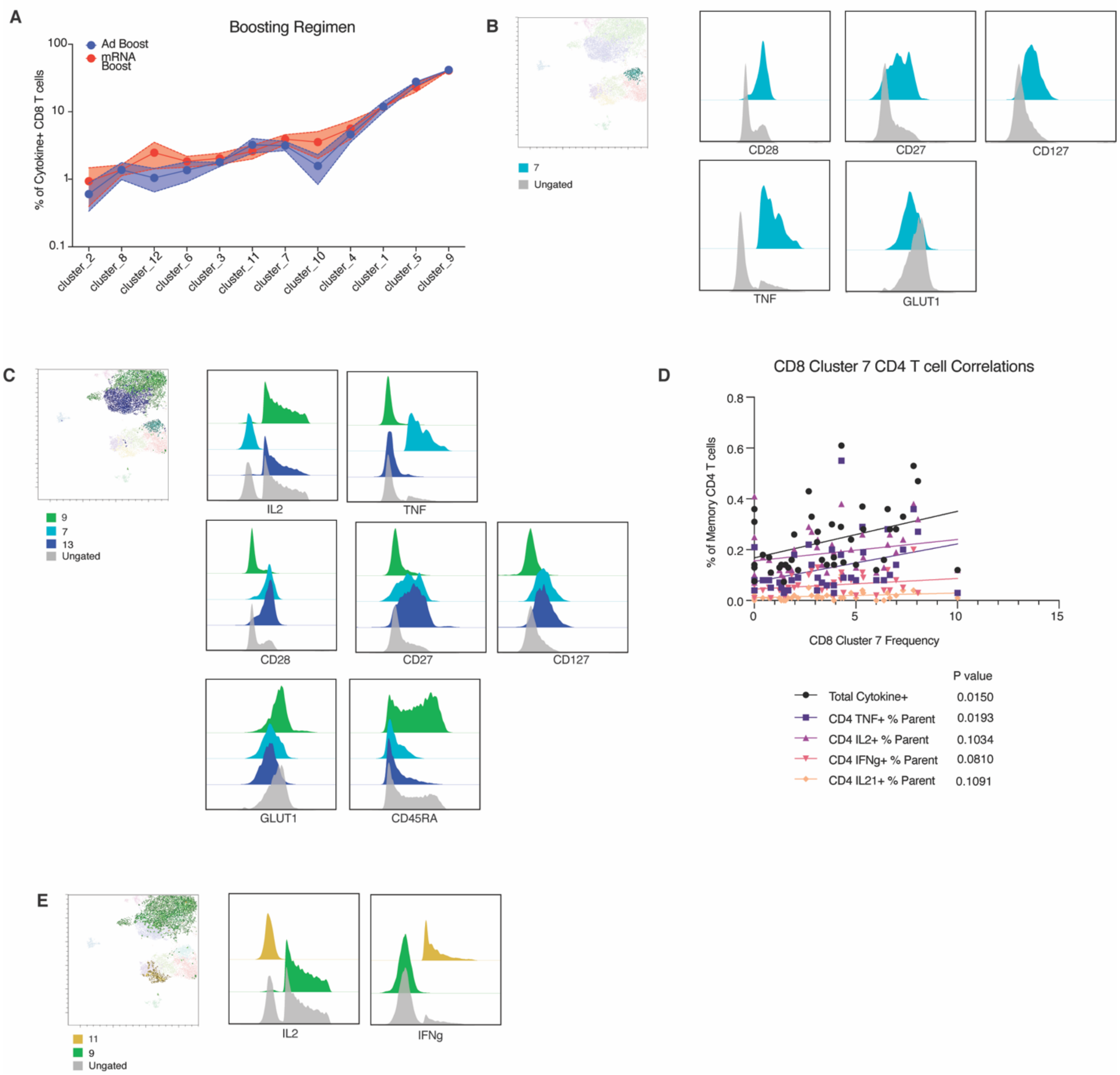

Figure S7: Phenotype of significant clusters identified in CD8 T cell compartment
UMAP projection of total cytokine-producing (i.e., producing IL-2, TNF, IFN-γ, or IL-21) memory CD8 T cells following overnight stimulation with BA.5 spike (S) peptides with Xshift clusters identified as significant highlighted. Histograms show markers that were most differentially expressed by indicated clusters. **A**. Frequency of clusters according to boosting regimen. Significance tested using repeated measures two-way ANOVA with the Geisser-Greenhouse correction. No significance detected **B**. Significant clusters according to individuals that mounted an antibody (Ab) response above the positive cutoff. **C**. Significant clusters according to individuals that mounted CD4 response above the average. **D.** Correlation of Cluster 7 frequency with memory T cells producing individual cytokines compared to total cytokine+ CD4 T cells. Correlation tested using non-parametric Spearman rank test. **E.** Significant clusters according to individuals that mounted CD8 response above the average.

Supplemental Table 1: Characteristics of SOTR cohort categorized by vaccine series.

Factor	PPP	MMM	PPJ	MMJ	p-value
N	15	20	21	19	
Days between dose 3 and lab draw, median (IQR)	14 (13, 16)	14 (14, 17)	15 (14, 16)	15 (14, 17)	0.60
Age at time of dose 1, median (IQR)	56 (44, 70)	56 (46, 66)	64 (50, 69)	65 (57, 72)	0.06
Age ≥65 at time of dose 1, n (%)	6 (40)	5 (25)	10 (48)	10 (53)	0.31
Female, n (%)	8 (53)	7 (37)	7 (33)	9 (47)	0.62
Race, n (%)					0.19
White	12 (80)	17 (85)	20 (95)	19 (100)	
Asian	1 (7)	0	0	0	
Hispanic, n (%)	2 (13)	1 (5)	1 (5)	0	0.36
Organ Allograft [a], n (%)					0.62
Kidney	11 (73)	10 (50)	14 (67)	13 (68)	
Liver	2 (13)	5 (25)	5 (24)	1 (5)	
Lung	0 (0)	1 (5)	0 (0)	1 (5)	
Heart	1 (7)	2 (10)	2 (10)	1 (5)	
Multi-organs	1 (7)	2 (10)	0 (0)	3 (16)	
Years between transplant surgery and dose 1, median (IQR)	2 (2, 7)	4 (2, 8)	7 (2, 15)	8 (3, 13)	0.19
≤5 years between transplant surgery and dose 1, n (%)	11 (73)	11 (55)	8 (38)	8 (42)	0.17
Calcineurin inhibitor, n (%)	15 (100)	16 (80)	17 (81)	15 (79)	0.24
Anti-metabolite, n (%)	12 (80)	14 (70)	17 (81)	18 (95)	0.27
mTOR inhibitor, n (%)	1 (7)	5 (25)	1 (5)	2 (11)	0.77
Belatacept, n (%)	0 (0)	1 (5)	1 (5)	2 (11)	0.77
Steroids, n (%)	8 (53)	9 (45)	12 (57)	14 (74)	0.33
Triple Immunosuppressant, n (%)	7 (47)	4 (20)	6 (29)	10 (53)	0.13

PPP: three homologous doses of Pfizer BNT162b2
MMM: three homologous doses of Moderna mRNA-1273
PPJ: two primary doses of Pfizer BNT162b2 followed by one dose of Johnson and Johnson/Janssen (JNJ) Ad26.COV2.S
MMJ: two primary doses of Moderna mRNA-1273 followed by one dose of JNJ Ad26.COV2.S
[a] organ allograft is mutually exclusive
[b] Triple immunosuppressant include calcineurin inhibitor, anti-metabolite, and steroid use

Supplemental Table 2: Crude and adjusted beta coefficient from multiple variable linear regression of CD4 T cell response of adenovirus compared to mRNA vaccine as a third dose.

T Cell Response	Crude β	p-value	95%CI		Adjusted* β	p-value	95%CI	
CD4 Cytokine (WT)	-0.100	<0.01	-0.169	-0.030	-0.091	0.01	-0.162	-0.019
CD4 Cytokine (BA.5)	-0.099	0.01	-0.174	-0.023	-0.095	0.02	-0.0175	-0.015
CD4 IL-21 (WT)	-0.012	0.03	-0.022	0	-0.010	0.054	-0.021	0
CD4 IL-21 (BA.5)	-0.006	0.23	-0.017	0	-0.007	0.25	-0.018	0.005
CD8 Cytokine (WT)	0.007	0.83	-0.056	0.069	0.016	0.62	-0.049	0.082
CD8 Cytokine (BA.5)	0.003	0.88	-0.042	0.049	-0.002	0.95	-0.049	0.046

*Adjusted for age ≥65 years old, ≤5 years post-transplant, liver-only transplant recipient, anti-metabolite, and belatacept use.

Supplemental Table 3. Flow Cytometry Panel

Specificity	Fluorochrome	Clone	Manufacturer
**Surface Markers**			
Viability	Live/Dead Blue	N/A	ThermoFisher
CD127*	PE-Cy5	HIL-7R-M21	BD Biosciences
CXCR3	BV650	1C6	BD Biosciences
CXCR5*	BB790	Custom	BD Biosciences
CCR7	BUV395	2-L1-A	BD Biosciences
CD28*	BV570	RF8B2	BD Biosciences
CD3	BUV496	UCHT1	BD Biosciences
CD4	BUV805	SK3	BD Biosciences
CD45RA	APC-H7	HI100	BD Biosciences
CD8α	BV510	HIT8a	BioLegend
KLRG1	PE-CF594	2F1	BD Biosciences
TCF1	Alexa Fluor 488	812145	R&D Systems
CTLA-4 (CD152)*	PE-Cy7	BNI3	BD Biosciences
OX40 (CD134)	BV711	ACT35	BD Biosciences
PD-1 (CD279)	BUV661	EH12.1	BD Biosciences
Tigit*	BB660	741182	BD Biosciences
TIM-3	BUV737	7D3	BD Biosciences
CD69	BV605	FN50	BD Biosciences
CD27	BV786	L128	BD Biosciences
**Intracellular Markers**			
Hexokinase II#	Dylight 680	EPR20839	Abcam
VDAC1^	Alexa Fluor 532	20B12AF2	Abcam
Tomm20	Alexa Fluor 405	EPR15581-54	Abcam
GLUT1	Alexa Fluor 647	EPR3915	Abcam
CPT1a#	PE-Cy5.5	8F6AE9	Abcam
IL-2	BV421	MQ1-17H12	BD Biosciences
TNF	BV750	MAb11	BD Biosciences
IL-21	PE	3A3-N2	BioLegend
IFNg	BB700	B27	BD Biosciences
FoxP3	Pacific Blue	206D	BioLegend

*Ordered as custom conjugate from BD Biosciences
#Self-conjugated using Abcam Lighting Link Conjugation kits (ab201804, ab102899)
^Self-conjugated using Thermo Fisher Alexa Fluor Antibody Labeling kit (A20182)
